# Biofilm-associated proteins: news from *Acinetobacter*

**DOI:** 10.1186/s12864-015-2136-6

**Published:** 2015-11-14

**Authors:** Eliana De Gregorio, Mariateresa Del Franco, Marianna Martinucci, Emanuela Roscetto, Raffaele Zarrilli, Pier Paolo Di Nocera

**Affiliations:** Dipartimento di Medicina Molecolare e Biotecnologie Mediche, Università Federico II, Via Sergio Pansini 5, Naples, 80131 Italy; Dipartimento di Sanità Pubblica, Università Federico II, Via Sergio Pansini 5, 80131 Naples, Italy

## Abstract

**Background:**

A giant protein called BAP (biofilm-associated protein) plays a role in biofilm formation and adhesion to host cells in *A. baumannii*. Most of the protein is made by arrays of 80–110 aa modules featuring immunoglobulin-like (Ig-like) motifs.

**Results:**

The survey of 541 *A. baumannii* sequenced strains belonging to 108 STs (sequence types) revealed that BAP is highly polymorphic, distinguishable in three main types for changes both in the repetitive and the COOH region. Analyzing the different STs, we found that 29 % feature type-1, 40 % type-2 BAP, 11 % type-3 BAP, 20 % lack BAP. The type-3 variant is restricted to *A. baumannii*, type-1 and type-2 BAP have been identified also in other species of the *Acinetobacter calcoaceticus-baumannii* (ACB) complex. *A. calcoaceticus* and *A. pittii* also encode BAP-like proteins in which Ig-like repeats are replaced by long tracts of alternating serine and aspartic acid residues. We have identified in species of the ACB complex two additional proteins, BLP1 and BLP2 (BAP-like proteins 1 and 2) which feature Ig-like repeats, share with BAP a sequence motif at the NH2 terminus, and are similarly expressed in stationary growth phase. The knock-out of either BLP1 or BLP2 genes of the *A. baumannii* ST1 AYE strain severely affected biofilm formation, as measured by comparing biofilm biomass and thickness, and adherence to epithelial cells. BLP1 is missing in the majority of type-3 BAP strains. BLP2 is largely conserved, but is frequently missing in BAP-negative cells.

**Conclusions:**

Multiple proteins sharing Ig-like repeats seem to be involved in biofilm formation. The uneven distribution of the different BAP types, BLP1, and BLP2 is highly indicative that alternative protein complexes involved in biofilm formation are assembled in different *A. baumannii* strains.

**Electronic supplementary material:**

The online version of this article (doi:10.1186/s12864-015-2136-6) contains supplementary material, which is available to authorized users.

## Background

*Acinetobacter baumannii* is a Gram-negative pathogen associated with multidrug resistance and hospital outbreaks of infection, particularly in the intensive care unit [1]. *A. baumannii* accounts for almost 80 % of all reported *Acinetobacter* infections, including ventilator-associated pneumonia, bacteremia, meningitis, peritonitis, urinary tract infections, and wound infections [1]. The rapid emergence of multidrug-resistant *A. baumannii* strains has resulted in limited treatment options, with most strains being resistant to clinically useful antibiotics [2]. *A. baumannii* cells readily form biofilms in vitro [3–5], and the ability of nosocomial strains to form biofilms on medical devices as in host tissues represents an important factor of microbial virulence. Cells forming biofilms are embedded within a polymeric conglomerate of proteins and polysaccharides. Biofilms are resistant to host immune defenses, detergents and antibiotics, and antibiotic resistance of microrganisms in these habitats can be increased up to a thousand-fold [6].

As in other microrganisms [7, 8] also in *A. baumannii* the formation of biofilm is a redundantly organized, multifactorial process involving multiple cellular components. The initial cell attachment to a surface is plausibly mediated by pili-like structures encoded by the *csu* locus [9], which are widespread among clinical isolates. However, the strong biofilm producer ATCC10696 strain lacks *csu*-encoded pili, as the ATCC17978 strain which expresses a different type of pili [10, 11]. Biofilms eventually grow by production of poly-beta-(1–6)-N-acetylglucosamine controlled by the *pga* locus [12]. The extracellular matrix provides adhesion between bacterial cells, enabling the formation of a multilayered structure. Several surface proteins are also involved in the process, and appear to differently contribute to the attachment of cells to biotic or abiotic surfaces. The major outer membrane protein OmpA is essential for the attachment of *A. baumannii* to human alveolar epithelial cells, but plays a role also in the development of biofilms on plastic [13]. In contrast, both cell adhesiveness and biofilm formation are high in *A. baumannii* isolates expressing the PER-1 extended-spectrum beta-lactamase [14].

Inactivation of a protein called BAP (for biofilm-associated protein) in the ST (sequence type) 1 AB307-0294 strain resulted both in decreased biofilm growth on glass [15] and decreased adherence to human bronchial cells [16]. BAP is expressed at the cell surface, and biofilm formation by BAP-positive strains is inhibited by affinity-purified BAP antibodies [17]. BAPs are large multidomain proteins playing a role in the process of biofilm formation both in Gram-negative and Gram-positive bacteria [18, 19]. These proteins exhibit poor sequence similarity, but share structural similarities, as they are internally repetitious and feature multiple (3 to 50 copies) immunoglobulin-like domains. These domains have a peculiar three-dimensional structure known as Ig fold, composed of 70–100 amino acid (aa) residues in seven anti-parallel beta-strands organized in two beta-sheets packed against each other in a sandwich structure [18]. *In S. epidermidis,* the Embp (extracellular matrix-binding protein) protein, involved both in cell adherence and biofilm formation [20], is enriched in modules different from Ig-fold repeats called FIVAR (Found In Various Architectures, 59 copies) and GA (G-related albumin-binding, 38 copies).

In different species, BAP genes are accessory genome components. The *E. faecalis* BAP gene is inserted in a 153 Kb pathogenicity island, the *S. aureus* BAP gene in a composite transposon comprising an ABC transporter operon and a transposase, in turn inserted in the 27 kb mobile pathogenicity island SaPIbov2 [21]. The gene was identified in 5 % of the *S. aureus* bovine mastitis, but in none of the human *S. aureus* isolates studied [22]. *S. epidermidis* isolates from animal mastitis forming biofilms possess a gene highly homologous to the *S. aureus* BAP gene, but not other SaPIbov2 sequences. Similarly, the Bhp (Bap homologue protein) protein is present only in some human *S. epidermidis* biofilm producers, as the RP62A strain [23].

In contrast, the BAP gene is largely conserved in the *A. baumannii* population, having been identified in isolates belonging to different STs [15]. Differently from other species, in *A. baumannii* the BAP gene is unlinked to genes involved in protein secretion. The BAP detected in *A. baumannii* ST92 isolates is approximately four times smaller than the AB307-0294 protein [17]. Size heterogeneity plausibly denotes changes in the number of protein repeats due to *recA-independent* slipped-strand mispairing during DNA replication, as reported for *S. aureus* BAP variants [24].

In this work, we have studied the organization of BAP coding sequences in wholly sequenced strains as in a large set of whole genome shotguns (WGS) of both *A. baumannii* (Additional file 1) and other *Acinetobacter* species. In silico analyses had been carried out also for two surface proteins, that we have called BLP1 and BLP2 (for BAP-like proteins) structurally related to BAP because similarly containing Ig-like domains. We showed that *A. baumannii* BAPs come in different formats, for changes in the number and type of repeats, and organization of the COOH region. Data mining suggest functional hierarchy linking BAP to BLPs, a hypothesis strengthened by the observation that gene disruption of either BLP gene impaired biofilm formation and adhesiveness to epithelial cells.

## Results

### Heterogeneity of BAP proteins among *A. baumannii* strains

The BAP protein identified in the *A. baumannii* strain AB307-0294 contains 8621 aa, and features seven distinct repeat units ranging in size from 70 to 104 aa. Repeats A-D exhibit poor similarity to each other, but all share Big_3_4 (Bacterial Ig-like domain, group 3) motifs, fitting the consensus TDnAGN, found in many bacterial surface proteins (PFAM accession n. PF13754). B, C and D repeats are over-represented, and account for 2/3 of the AB307-0294 BAP. Repeats E, F and G lack Big_3_4 motifs, and are reiterated in tandem in the COOH region. A third G-like repeat is located downstream at 170 aa distance. A-G repeat sequences are reported in Additional file 2.

**Fig. 1:**
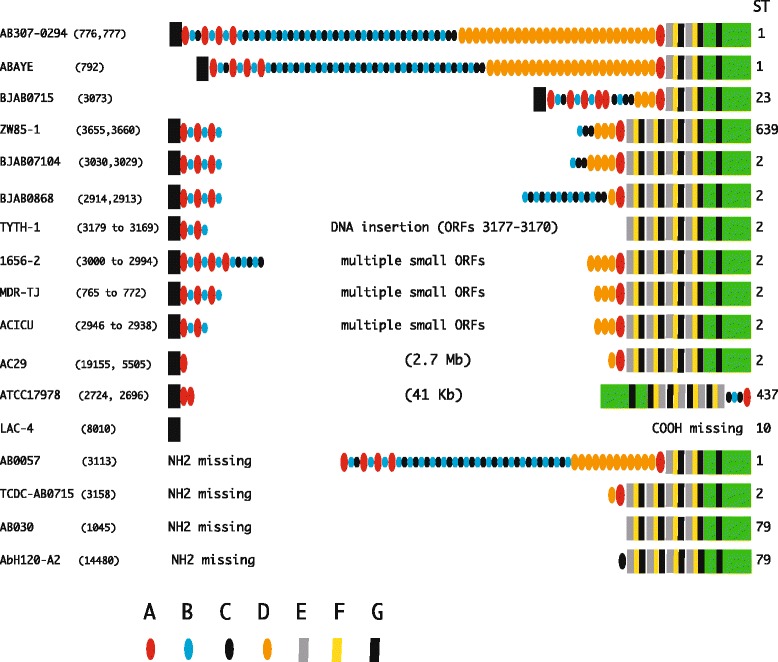
Organization of BAPs coding sequences in wholly sequenced *A. baumannii* genomes. The organization of coding sequences homologous to BAP [15] in wholly sequenced *A. baumannii* genomes deposited at the KEGG (Kyoto Encyclopedia of Genes and Genomes) site is depicted. Boxes denote NH2 and COOH regions, ovals modules of the repetitive region. Modules within small ORFs spanning the repetitive region in different clones have not been reported for clarity. Numbers in parenthesis denote ORFs, numbers to the right of each protein the strain ST

In BAP proteins found in wholly-sequenced *A. baumannii* genomes (Fig. 1), the NH2 region is conserved, the COOH region varies in length because either 2 or 4 copies of the EFG module are present (Additional file 2). Small and large COOH regions, which exhibit 44 % sequence identity, mark BAPs encoded by strains belonging to the abundant sequence types ST1 and ST2, which correspond to international clone I and II, respectively [25]. Accordingly, the corresponding proteins have been classified as type-1 and type-2 BAPs. Length and composition of the central repetitive region is also variable. According to GenBank annotations, intact BAPs are encoded by the AYE (8200 aa) and BJAB0715 (3059 aa) strains only, while the 8621 aa AB307-0294 protein described by Loehfelm and coworkers [15] is split in ORFs 776 and 777. The discrepancy may either signal that a mutation occurred in the AB307-0294 BAP gene prior to whole-sequencing, or denote sequencing errors. In this context, it may be worth noting that the *Salmonella thyphimurium* LT2 strain encodes a 386 kDa BAP, but the corresponding gene is annotated in GenBank as a pseudogene because of a frameshift mutation [26]. In other *A. baumannii* genomes, BAP homologous sequences are split in two or more ORFs (six in strains 1656–2, MDR-TJ and ACICU). It is unlikely to hypothesize in all instances that the fragmentation reflects sequencing errors, sequencing data having been obtained by different investigators. In several ST2 strains (ZW85-1, BJAB0714, BJAB0868, MDR-TJ, MDR-ZJ06), BAP prematurely terminates at residue 737. The corresponding COOH region, preceded by a variable number of repeats, is encoded by downstream flanking ORFs. This finding and the size identity of small BAP ORFs in MDR-TJ and ACICU strains, both support the notion that allelic BAP-negative variants, spread by horizontal gene transfer, may be common in the bacterial population. In the ATCC17978 and A12 strains, the BAP gene was disrupted by a 7 Kb DNA insertion in TYTH-1, and by chromosomal rearrangements moving away, or flipping the NH2 and COOH regions. In six strains, either the NH2 or the COOH region is missing, in AC29 the two regions are at 2.7 Mb distance (Fig. 1).

### BAP in *A. baumannii* draft genomes

Data in Fig. 1 suggest that BAP may be an accessory, dispensable gene expressed by subsets of the *A. baumannii* population only. To learn more, BAP homologs were searched by tBLASTn in a large set of draft genomes of *A. baumannii*. MLST analyses were performed at the A. *baumannii* MLST website (http://pubmlst.org/abaumannii/) using the “Pasteur” MLST scheme [25]. Only strains which could be assigned to an ST were selected for further studies. The degree of identity of rpoB gene sequences helped to reassign several strains annotated as *A. baumannii* to other species. On the whole, the *A. baumannii* strains analyzed in this work (both complete and draft genomes) are 541 and belong to 108 different STs (Additional file 1). For sake of simplicity, hereon draft genomes and/or the corresponding strains will be referred to by the four-letter code in the GenBank accession number (see Additional file 1).

Intact BAP coding sequences have been identified only in the AYOI (6207 aa) and JEVZ (6219 aa) strains. In a few high quality draft genomes (AFDB, AFDK, AYOH, JFEL, JFXM), BAP coding sequences resided in one contig, but were interrupted by stop codons, and split into two or multiple ORFs, as in many wholly sequenced genomes shown in Fig. 1. In the remaining WGS, sequences encoding NH2 and COOH BAP regions, flanked by segments of the central repetitive region of variable lengths, were at the termini of two contigs. The stop codon at BAP residue 738 found in several wholly-sequenced genomes is a signature conserved in 42 % (118/282 genomes) of the ST2 strains analyzed. Given the prevalence of ST2 strains, interrupted BAP genes may be frequent in the *A. baumannii* population.

The NH2 and COOH regions were conserved in most of the clones analyzed. Changes in the region encompassing the terminal 397 aa mark BAP-2b and BAP-2c variants which exhibited 46 and 41 % identity to BAP-2 in the diversity region, respectively. BAP-2b have been found in all the 12 ST499 strains analyzed and two ST2 strains (AFTC and AMSX), BAP-2c in the single ST35 APRA and the ST504 JEXU strains.

**Fig. 2:**
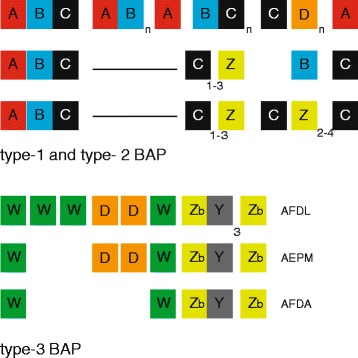
Repeat modules in type-1, type-2 and type-3 BAPs. Bars denote undefined repeat regions in Z-plus type-1 and type-2 BAPs. AFDL, AEPM and AFDA clones potentially express a complete type-3 BAP

Changes in the composition of the BAP repetitive region were also observed. A novel Ig-like Z repeat unrelated to A-D repeats was identified in 69 strains belonging to 26 different STs. As sketched in Fig. 2, the overall module organization in Z-positive and Z-negative regions differs. Type-3 BAPs have the same NH2 region of type-1 and type-2 BAPs, but differ both in the repetitive and the COOH regions. The repetitive region features D, Zb (a Z variant restricted to type-3 BAPs), W and Y modules (Fig. 2,; the sequence of the modules are in Additional file 2). The COOH region (1169 aa) of type-3 BAP exhibits only 40 % identity to the corresponding region of type-1 or type-2 BAPs. EFG modules are absent, but a tract of approximately 60 aa, exhibiting 50 % identity to G repeats, could be identified. The 26 clones carrying type-3 BAPs belong to 14 different STs, and about half of them belong to ST25. Type-3 BAPs are relatively limited in size, the largest complete protein found in the AFDL strain measuring 2700 aa (Additional file 2). Here too, however, it must be emphasized that the complexity of the repetitive region might have been missed in sequencing assembly.

On the whole, strains encoding type-1, type-2 and type-3 BAP belong to 29, 40 and 11 % of the STs analyzed. The remaining STs lack BAP, or carry truncated forms of the protein.

### BAP-like proteins encoded by the *A. baumannii* SDF strain

The *A. baumannii* SDF strain, isolated from a human body lice [27] encodes two unusual BAP-like proteins, ABSDF785 (2321 aa) and ABSDF2314 (2402 aa), which exhibit significant homology to BAPs at the NH2 terminus only. In both, Ig-like repeats are replaced by long stretches of alternating serine (S) and aspartic acid (D) residues (501 and 701 SD repeats in ABSDF785 and ABSDF2314, respectively).

### BAPs in other *Acinetobacter* species

BAP proteins occur in the genomes of several non*-baumannii Acinetobacter* strains (Additional file 3). The list includes the *A. baylyi* ADP1, all the strains classified as *A. calcoaceticus* (taxid 471), *A. pittii* (taxid 48296), and *A. radioresistens* (taxid 40216), as several *A. haemolyticus* (taxid 29430) and *A. nosocomialis* (taxid 106654) strains, only one (APPX) of the six strains classified as *A. junii* (taxid 40215) BAP proteins are missing in *A. johnsonii* and *A. lwoffii*.

**Fig. 3:**
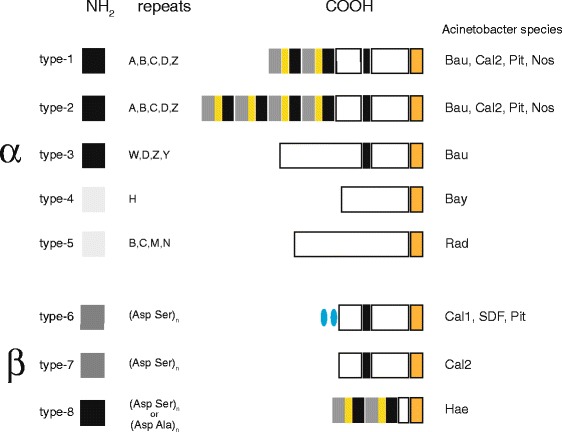
Alpha and beta BAPs. Changes in the organization of the 8 BAP types found in the Acinetobacter genus are highlighted. Bau, *A. baumannnii*; SDF, *A. baumannnii* SDF; Hae, *A. haemolyticus*; Pit, *A. pittii*; Nos, *A. nosocomialis*; Bay, *A. baylyi*, Rad, *A. radioresistens*, Cal, *A. calcoaceticus.* Cal1 and Cal2 refer to the wholly sequenced *A. calcoaceticus* PHE-A2 [41] strain and the draft genomes deposited at the NCBI, respectively. Sequences conserved at the end of all BAP types are in orange. For sake of simplicity difference in the COOH regions among BAPs are not highlighted. E, G, F and B modules are depicted as in Fig. 1

*Acinetobacter* BAPs have been classified as alpha or beta for the presence of either Ig-like or SD repeats as repetitive modules, respectively, and assigned to 8 types on the basis of their COOH regions (Fig. 3). In addition to *A. baumannii*, type-1 and type-2 BAPs have been found in *A. calcoaceticus*, *A. pittii* and *A. nosocomialis*, while type-3 BAPs are confined to *A. baumannii. A. baylyi* and *A. radioresistens* BAP-like proteins markedly differ from *A. baumannii* BAPs because the repetitive region features either highly divergent copies of a single Ig-like module (type-4), or novel Ig-like modules interrupted by foreign aa stretches (type-5) (Additional file 4).

Type-6 and type-7 beta-BAPs differ for the presence/absence of B modules in the repeat region. Curiously, *A. pittii* and *A. calcoaceticus* strains producing beta-BAPs potentially encode also alpha type-1 BAPs. *A. haemolyticus* type-8 BAPs are hybrid alpha/beta proteins which resemble type-1 BAPs in the COOH region, but feature long tracts of alternating aa in the repetitive region. The complete proteins encoded by the *A. haemolyticus* ATCC19194 and TG19602 strains feature 79 SD pairs and 148 AD pairs, respectively.

The NH2 region is largely conserved in all BAPs, but three main variants, denoted by the color code in Fig. 3, could be recognized (Additional file 5). Curiously, the *A. baumannii* and *A. haemolyticus* NH2 regions both feature a 38 aa insertion mostly made by tandem DA repeats. Significant similarities of the COOH regions of the various BAP types are limited to the terminal 100–150 aa. It may be worth noting that only G modules are conserved in many BAPs (Additional file 5).

The analysis of wholly sequenced genomes showed that alpha- and beta-BAP genes map at different chromosomal sites. The alpha gene region is conserved in *A. baumannii*, *A. calcoaceticus* PHE-A2 and *A. baylyi ADP1* genomes. In the *A. baumannnii* SDF strain, the region is also conserved, but is occupied by the beta-BAP ABSDF785 gene. The beta gene region is located on genomic islands, flanked by *lap*EBC genes coding for components of a type-I secretion system, both in *A. calcoaceticus* PHEA-2 and *A. baumannii* SDF strains (Additional file 6). SD-rich adhesion protein genes flanked by *lap* genes have been found in different Gram-negative microrganisms, such as *Klebsiella pneumoniae* MGH 78578 (ORF KPN_00994), and *Enterobacter aerogenes* EA1509E (ORF ST548_p6177).

### Additional proteins with Ig-fold motifs encoded by *A. baumannii*

**Fig. 4:**
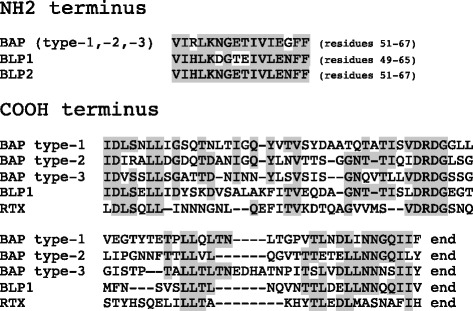
Conserved motifs in BAP, BLP1 and BLP2. Sequence motifs conserved at the termini of the three proteins are highlighted. RTX refers to the 1450 aa RTX toxin (ORF1891) encoded by the *A. baumannii* ACICU strain

In *A. baumannii*, two additional proteins were found to contain Ig-like repeats. The proteins, variously named in different annotations, have been named, for sake of simplicity, BLP1 and BLP2. Both BLPs and BAP share a sequence motif at the NH2 terminus. BLP1 and BAP feature COOH-terminus aa sequences similar to those found in RTX toxins, proteins secreted through a type I secretion system ([28]; see Fig. 4). In the AYE strain, BLP1 and BLP2 correspond to ORFs 821 and 1037, respectively, and BAP to ORF 792. BAP and BLP1 genes are thus very close (approximately 30 kb away), and their relative position is conserved in all strains examined.

**Fig. 5:**
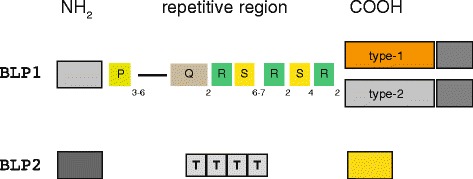
*A. baumannii* BLP1 and BLP2 proteins. The modular organization of the two proteins is diagrammed. The 297 aa “unique region” between P and Q repeats in BLP1 is depicted by a bar

BLP1 and BLP2 exhibit the same tripartite structure of BAP (Fig. 5). In BLP1, the repetitive region is made up by four different repeat types (P, Q, R, S), all but Q featuring Big_3_4 motifs (Additional file 7). BLP1 vary in length from 3044 to 3356 aa. BLP1 has a small number of repeats, and complete proteins have been identified in about 60 % of the examined WGS. Aminoacids changes let to distinguish type-1 and type-2 BLP1 variants, exhibiting 86 % identity in a 552 aa domain of the COOH region. The nomenclature reflects the occurrence of type-1 and type-2 BLP1 in clones of the predominant ST1 and ST2 types. Type-2 variants featuring a more extensive remodeling of the variable domain are encoded by a few strains, each belonging to a different ST, and have been named H and K variants (see Additional file 7).

BLP2 features a simple repetitive region made by only four T repeats, and a relatively small (207 aa) COOH region. The protein is conserved in most of the strains examined, and four main sequence variants (A-D) could be recognized. The A-type has been found in >70 % (77/108) of the STs examined, including the predominant ST1 and ST2. The minor B, C and D variants, which exhibit decreasing similarity to the A-type protein, have been found in 11, 13 and just 2 STs, respectively. In both C- and D-type proteins, the Ig-like domain of one T repeat is mutated (Additional file 7).

In contrast to BLP2, BLP1 had been identified only in 80 % of the clones analyzed. BLP1-negative strains fall into two distinct groups. In the JFXH, APWV and ATGJ strains, the BLP1 coding region had been completely or partly deleted. In the majority of BLP1-negative strains, however, the BLP1 coding region is invariably replaced by a 99 bp DNA tract (Additional file 8).

### BLPs in other *Acinetobacter* species

BLP1 and BLP2 genes were searched in non*-baumannii Acinetobacter* genomes (Additional file 3). *A. calcoaceticus* and *A. pittii* strains encode both BLPs, *A. haemolyticus* and *A. nosocomialis* strains BLP2 only. In all the BLP1-negative species, sequences homologous to the 99 bp DNA tract found in the *A. baumannii* BLP1-negative clones could be identified (Additional file 8). The presence/absence of BLP1 in the *A. baumannii* population can thus be rationalized by hypothesizing that the BLP1 gene region had been replaced by the corresponding “empty site” upon recombination with non-*baumannii* cells. Exchange of the BLP1 locus among *Acinetobacter* cells is frequent, as indicated by the spread in *A. baumannnii* of the H and K BLP1 variants found in *A. calcoaceticus* and *A. pittii* strains.

*A. johnsonii*, *A. lwoffii* and *A. junii* strains lack both BLP1 and BLP2. In 5/6 *A. junii* genomes analyzed, BLP2-like genes are inactivated by a frameshift mutation, separating NH2 and COOH regions into two ORFs, and the BAP gene is missing. Only the *A. junii* APPW strain encodes an intact BLP2, and is worth noting that only this strain potentially encodes also a BAP protein.

### Sequence type distribution of BAP, BLP1 and BLP2

The distribution of BAP, BLP1 and BLP2 among analyzed STs is summarized in Additional file 9. A close look at data support the notion that the three proteins may be functionally related. BLP1 seems ancillary to BAP, since it is missing in almost all the STs (14/16) which lack BAP. Among BAP-positive strains, the BLP1 gene is unevenly distributed. BLP1 was found in most (42/47) STs expressing a type-2 BAP, 60 % of STs expressing a type-1 BAP, only in one of the 12 STs expressing a type-3 BAP. Moreover, type-2 BAP and type-2 BLP1 are more frequently associated than type-1 BAP and type-1 BLP1. Thus, the coexistence of some BLP1 and BAP variants may be preferred, while specific combinations may plausibly be disadvantageous, and this may explain the exchange of filled/empty BLP1 sites observed in the population. BLP2 is conserved in most of the clones examined. Several BAP-negative STs lack BLP2, others feature BLP2 variants which may be functional impaired because carrying mutations in one Ig-like domain (Additional file 9), suggesting that BAP and BLP2 may be interdependent. The hypothesis that BAP and BLP2 may functionally interact was reinforced by the analysis of *A. junii* genomes. BAP and BLP2 are absent in all *A. junii* strains examined but the APPW strain, in which both proteins are present.

Functional link of BAP, BLP1 and BLP2 was further provided by the results of quantitative RT-PCR analyses in which the levels of the corresponding transcripts were monitored. For all, RNA levels were undetectable in log-phase, high in late stationary phase (data not shown).

### Biofilm formation in different *A. baumannii* strains

**Fig. 6:**
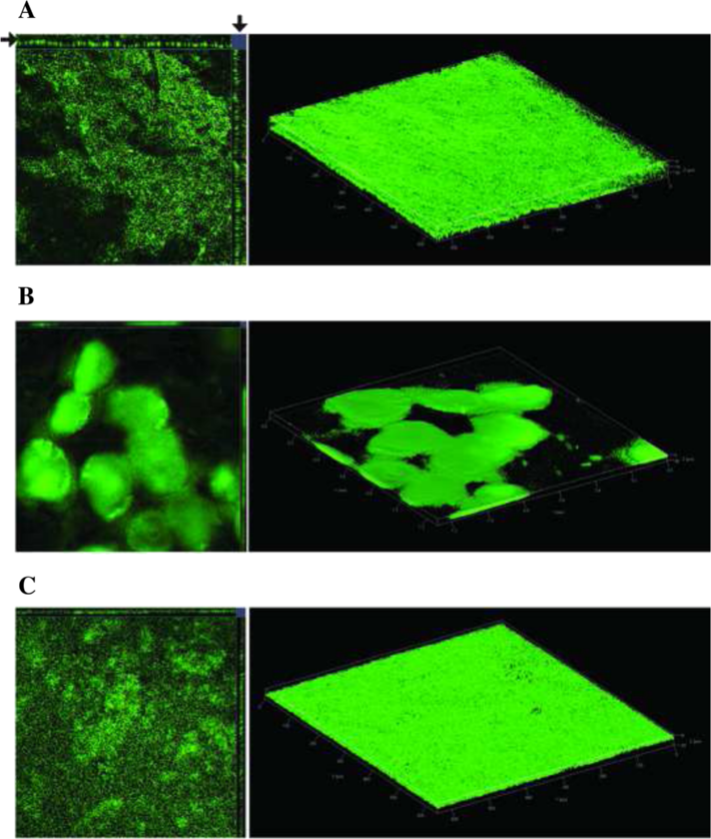
CLSM analysis of biofilms formed by the *A. baumannii* strains ACICU (**a**), 4190 (**b**), and ATCC17978 (**c**). In each panel, to the left is shown the orthogonal view of Z-stacks, to the right the three-dimensional spatial distribution of the biofilm. Arrows denote biofilm heights. Bright and dark areas show cell clusters and voids in the biofilm

**Fig. 7:**
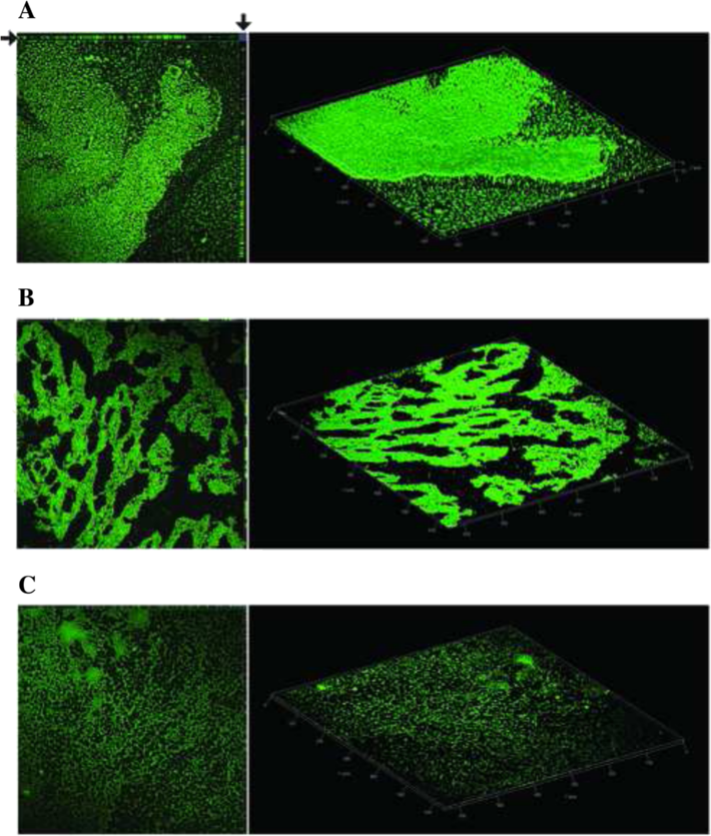
Biofilms CLSM analysis. Biofilms formed by the AYE (**a**), the AYE-ΔBLP1 (**b**) and AYE-ΔBLP2 (**c**) strains. Orthogonal view of Z-stacks, three-dimensional spatial distribution of the biofilm and arrows are as in Fig. 6

**Fig. 8:**
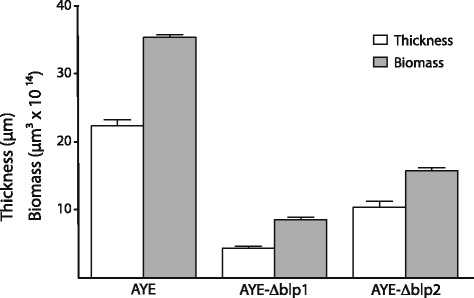
Quantitative analysis of biofilm formation. The thickness (*white bars*) and the biomass (*grey bars*) of biofilms formed by the *A. baumannii* AYE, AYE-ΔBLP1 and AYE-ΔBLP2 strains are shown

We thought of interest to evaluate differences in the type and extent of biofilm formation among *A. baumannii* strains belonging to different STs, and presenting a different combination of BAP and BLP1 proteins by CLSM (Confocal Laser Scanning Microscopy) analyses. The ACICU (type-2 BAP, BLP1-plus) and the 4190 (type-3 BAP, BLP1-negative) strains, assigned to international clone II and ST25 epidemic clonal lineage, respectively, were shown to be relatively strong biofilm producers [4]. Both strains carry *pga*, *csu* and *omp*A genes shown to be involved in biofilm production. Biofilms formed by the two strains have comparable heights, but different architectures (Fig. 6, panels a and b). The ACICU biofilm exhibited a total substratum coverage. By contrast, the 4190 biofilm was made up by large, evenly distributed microcolonies, separated by water channels. The ATCC17978 strain also carries *pga*, *csu* and *omp*A genes, but lacks both BLP1 and BAP1. Yet, this strain formed a dense biofilm, exhibiting an uniformely surface coverage. However, microcolonies were undetectable (Fig. 6, panel c). The heterogeneity of the biofilms formed by the analyzed strains is not surprising. The plethora of genes shown or hypothesized [11] to be involved in biofilm formation in *A. baumannii* makes difficult evaluating the role that different BAP types, and the presence/absence of BLP1, may play in the process. We thought therefore to address the problem by mutating, as done for the BAP gene in the B307-0294 strain [15], the BLP1 and BLP2 genes of the AYE strain. Either gene was inactivated by allelic replacement [29]. The test strain encodes a sequence-proved intact BAP, comparable in length and composition to the B307-0294 protein (see Fig. 1). BAP and BLP1 expressed by the AYE and B307-0294 strains, which both belong to sequence type 1, are functionally comparable, because unaffected by size changes which may influence their exposure on cell surface, as proved for repetitive *S. aureus* SDR proteins of different lengths [30]. The biofilms formed on glass by AYE and its BLP1 deletion derivative AYE-Δblp1 markedly differed. AYE formed a typical biofilm structure (Fig. 7a), in which distinct microcolonies could be distinguished. In contrast, AYE-Δblp1 formed just a thin monolayer. The width of the bioflm formed by AYE averaged 22 microns. The biofilm formed by the mutant (average 4 microns) was barely measurable (Fig. 7b). Inactivation of BLP2 also affected biofilm formation (Fig. 7c). The AYE-Δblp2 mutant produced a biofilm only twice thinner than AYE. However, in contrast to the AYE-Δblp1 biofilm, the coverage was reduced, and microcolonies could not be detected. Quantitative estimates obtained using the IMARIS v7.0 software (Bitplane, Switzerland) allowed measuring the total biomass of biofilms as micrometers^3^ [31]. Quantitative estimates of the biofilms formed by AYE, AYE-Δblp1 and AYE-Δblp2 are shown in Fig. 8. A significant correlation was found between biofilm biomass and biofilm thickness of AYE and AYE-Δblp1 and AYE-Δblp2 mutant strains (*r* = 0.9974, *p* = 0.0455).

### Adherence of *A. baumannii* wild type AYE and AYE-Δblp1 and AYE-Δblp2 mutant strains to A549 human bronchial cells

**Fig. 9:**
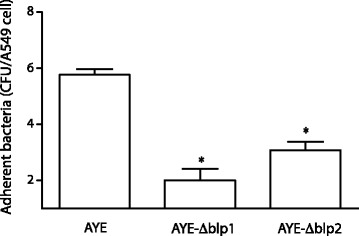
Bacterial adherence of *A. baumannii* AYE, AYE-ΔBLP1 and AYE-ΔBLP2 strains to A549 bronchial epithelial cells. Cell surface-associated bacteria after 60 min incubation at 37 °C. Asterisks denote statistically significant (*p* <0.001) differences in the degree of cell adhesion of AYE and derivative clones

We next investigated the ability of AYE, AYE-Δblp1 and AYE-Δblp2 strains to adhere to A549 human alveolar epithelial cells. As shown in Fig. 9, AYE showed a statistically significant higher adherence to A549 human bronchial cells compared with AYE-Δblp1 and AYE-Δblp2 mutant strains, which showed a reduction in adherence by 2.9- and 1.7 fold, respectively. Moreover, significant correlations were found between adhesiveness to epithelial cells and biofilm biomass (*r* = 0.9999, *p* = 0.0104) and bacterial adhesiveness to epithelial cells and biofilm thickness (*r* = 0.9985, *p* = 0.0351) for AYE, as for AYE-Δblp1 and AYE-Δblp2 mutants. On the other hand, AYE and mutant strains were not able to invade A549 cells human alveolar cells (data not shown). Also, a similar number of bacteria adhered to A549 cells when the monolayers were incubated with *A. baumannii* strains for 60 min at 4 °C, i.e. under conditions that do not allow for tissue invasion (data not shown).

### Variable assortment of other surface proteins

We checked next whether the distribution of other surface proteins may similarly vary among *A. baumannii* strains. OmpA, the abundant 356 aa protein involved in biofilm formation [13] is highly conserved (>95 % identity) in most isolates. In 16 clones, however, a protein variant exhibiting only 70 % identity at the NH2 side (residues 35–183 aa) was detected (Additional file 10). Interestingly, all these clones but one potentially expressed a type-3 BAP. SURP-1 (SUrface repetitive protein 1) is a 904 aa protein with a central region made by 84–93 aa repeats (Additional file 10) unrelated to either BAP or BLP1. SURP-1 has been found only in 65 % of the ST. The protein is missing in all STs featuring type-3 BAP, except ST 215, 50 % of BAP-negative STs, most of the BAP-plus/BLP1-negative STs. The distribution of OmpA and SURP-1 among the various STs is summarized in Additional file 9.

## Discussion

Most bacterial species form biofilms, and the identification of the genetic determinants controlling this complex process became an active field of research in recent years, given the implications that biofilm formation has in medical care. As in other pathogens, a large protein expressed at the *A. baumannii* cell surface, known as biofilm-associated protein or BAP, has a role in biofilm formation as in host cell adherence [15, 17]. Comparative genome analyses carried out over hundreds of sequenced strains revealed that the *A. baumannii* BAP is highly polymorphic. The central repetitive region has a variegated structure, and type and number of repeat modules both vary among isolates. This region connects a relatively small NH2 domain, conserved in all BAPs which is plausibly exposed on the cell surface and involved in binding, to large COOH regions in turn hosting a few, additional repeats. BAP COOH regions also vary, and three main types of proteins could be distinguished. Type 1 and type 2 proteins are expressed by strains of the predominant clonal complexes 1 and 2 strains, respectively, and have been identified also in *A. calcoaceticus*, *A. pittii* and *A. nosocomialis*, which form with *A. baumannii* the monophyletic ACB complex [32, 33]. Type 3 BAPs feature short repetitive regions, which include novel repeat modules, and a COOH region exhibiting only 40 % similarity to the corresponding regions of either type-1 or type-2 BAPs. This protein variant seems restricted to *A. baumannii*. However, taking into the account that the variant had been identified only in 13/108 STs, and a relatively small number of non*-baumannii Acinetobacter* strains had been sequenced, it cannot be ruled out that type 3 BAP may be expressed also by other species of the ACB complex.

The genus *Acinetobacter* hosts a variety of BAP proteins, all featuring large central repetitive regions made either by 80–100 aa long Ig-like repeats (alpha-type BAP), or homopolymeric stretches of alternating serine and aspartic acid, or serine and alanine residue containing up to 500 dipeptides (beta-type BAP). Repetitive regions in both alpha- and beta-type BAPs span the cell-wall allowing exposure of the N-terminal region to the environment [15, 30, 34]. The length of the repeat region has been shown to influence exposure of the *S. aureus* SDR proteins on cell surface [30], and the same may plausibly hold true for alpha-BAPs. In alpha-like BAPs found in other species, the repeat region originated by the expansion of a single module ([35, 36]). The rule is subverted in *A. baumannii* BAPs, which feature multiple repeats varying in length, composition and relative arrangement. A variegated repetitive region may be beneficial for such a large protein, which is a gain of specific isolates in many species, because encoded by mobile DNA, but is in contrast a conserved gene product in *A. baumannii*. Modules diversification should decrease the chance that the protein may vary in size because of strand slippage during DNA replication. Moreover, a low sequence identity between modules strongly inhibits misfolding and aggregation of repetitive proteins, and consecutive homologous domains in large multi-domain proteins almost exclusively show sequence identities of less than 40 % [37].

As shown in this report, differences in size and extent of module variation among *A. baumannnii* BAPs both remain largely unknown. The number of isolates in which the BAP gene is functional, or had been inactivated by mutations within the repetitive region, as reported for different genomes (see Fig. 1), remains also to be established, and the issue may be settled for individual strains only by expression analyses. Mapping repetitive DNA regions is a major bias in whole sequencing projects. Reconstructing the organization of the baroque BAP repetitive region is a tremendous, pitfall-rich task, and most of the sequence data on *A. baumannnii* BAP could be wrong, and need to be amended. Translation of the BAP gene RNA is interrupted at residue 737 of the protein in a high number of ST2 clones, but a −1 frameshifting could let translation extending into downstream BAP coding sequences. While the occurrence of both short and long BAP isoforms in these clones may be experimentally tested, data denote variation in the state of the BAP gene among strains of the same ST. In many isolates, the BAP is truncated, or absent. Altogether, the fraction of BAP-negative isolates belonging to different STs is elevated (Additional file 9). Thus, the BAP gene may be catalogued as a variable dispensable gene.

In *A. baumannii*, two additional proteins, BLP1 and BLP2, include a central repetitive region made by Ig-like repeats. BAP, BLP1 and BLP2 share a large sequence motif at the NH2 terminus, are co-expressed at the end of the stationary growth phase, and may work in conjunction. BLP1 is a large (3044 up to 3356 aa) protein which features a composite repetitive region, and can be typed, as BAP, into main sequence variants according to aa changes in the COOH region. BLP1 has been found in *A. baumannii*, *A. calcoaceticus* and *A. pittii.* In several *A. baumannii* isolates, the BLP1 coding region is missing, and is invariably replaced by the same piece of DNA which is highly homologous to DNA tracts found, at the same relative chromosome position, in *A. nosocomialis* and other *Acinetobacter* BLP1-negative species. The coexistence of empty and filled BLP1 sites in the population implies recombination of *A. baumannii* with non*-baumannii Acinetobacter* cells. The genetic exchange plausibly occurred once, and the empty site status had been eventually maintained, probably because the absence of BLP1 turned out to be advantageous in specific genetic milieu. The hypothesis stems from the uneven distribution of BLP1, conserved in most isolates expressing type-2 BAP, but only in half of those expressing type-1 BAP, missing in isolates expressing type-3 BAP. BLP1 is also missing in most BAP-negative isolates. Altogether, data suggest that BAP and BLP1 may be interdependent. The proteins may interact and/or share a partner on the cell surface, and the combination of BLP1 with different BAP types may be beneficial or harmful. BLP2 is a smaller (728 aa) protein with a repetitive region made by only 4 Ig-like repeats. The protein, found in all the species of the ACB complex, is highly conserved in *A. baumannii*. BLP2 may also be a component of surface protein complexes involved in biofilm formation. The conclusion stems from the observation that BAP-negative strains belonging to different STs lack BLP2, or feature BLP2 with mutated Ig-like domains. Moreover, BAP and BLP2 are both absent or present in all *A. junii* strains examined.

A link between BAP and BLPs is suggested by the observation that gene disruption of either BLP gene impaired biofilm formation, as proved by SCLM analyses (Figs. 7 and 8). Similarly to BAP, the BLP1- and BLP2-negative derivatives AYE-Δblp1 and AYE-Δblp2 also showed a reduced adherence to human epithelial cells (Fig. 9). Other surface proteins, known or hypothesized to be involved in biofilm formation, may interact and/or be assembled with BAP and BLPs on the cell surface. OmpA has a role in biofilm formation [13] and is highly conserved in the *A. baumannii* population. Interestingly, the protein varies significantly in sequence composition in most clones expressing type-3 BAP. SURP-1, a repetitive protein structurally related to BAP and BLPs, is missing in most type-3 BAP, as in many BLP1-negative clones. The uneven distribution of the various BAP types, OmpA variants, BLP1 and SURP-1 proteins among isolates is indicative of alternative combinations of surface proteins in the *A. baumannii* population. Consequently, the architecture of the biofilm formed by different isolates may greatly vary.

## Conclusions

The construction of clones expressing BAP and BLPs isoforms in all possible combinations is important to clarify the role played by each protein in biofilm formation of *A. baumannii*, establish whether the proteins interact, assess underneath hierarchies. In parallel, mutational analyses may shed light on the functional organization of the repetitive regions of BAPs and BLPs, and establish how the combination of specific modules may influence secretion and surface exposure of each protein.

The assay of type-3 BAP derivatives may be crucial to assess whether the unicity of this protein, which seems mutually exclusive relative to other surface components, correlates with the particular combination of Ig-like modules in the repetitive region and/or their paucity, or resides in the COOH region.

## Methods

### In silico data

Tblastn searches of BAP and BAP-like proteins in *A. baumannii* and other *Acinetobacter* species WGS were carried at the National Center for Biotechnology Information (NCBI) (http://www.ncbi.nlm.nih.gov/). Modules in BAP repetitive regions were marked according to the AB307-0294 BAP protein nomenclature ([15]; accession number EU117203).

Tandem repeats in BAP, BAP-like and other repetitive proteins were identified by using the XSTREAM (variable Sequence Tandem Repeats Extraction and Architecture Modeling, [38]) and the RADAR (Rapid Automatic Detection and Alignment of Repeats, [39]) algorithms. Searches are restricted to Whole Genome Shotgun (WGS) genomes deposited at GenBank before March 2014.

### Construction of AYE-Δblp1 and AYE-Δblp2 mutants

The genes encoding the BLP1 and BLP2 proteins of the *A. baumannii* AYE strain [27] were mutagenized by allelic replacement, according to the published procedure [29]. Flanking fragments located immediately upstream and downstream from the AYE *A. baumannii* BLP1 and BLP2 genes were amplified by PCR and cloned into the suicide vector pMo130-TelR, which contains the tellurite-resistance marker and the sacBR genes conferring sucrose sensitivity [29]. Upstream and downstream BLP1 and BLP2 fragments were amplified using the pairs of BLP1 and BLP2 up and dw primers shown below:

- BLP1up-fw: tatgcggccgcaacagcctgaagtgattgttgtg (*NotI* site underlined)
- BLP1up-rv: gctggatccttaatatcaatttttgcgattaccttc (*BamHI* site underlined)
- BLP1dw-fw: tatggatcctagacgaattactaaacaaccagca (*BamHI* site underlined)
- BLP1dw-rv: tatgcatgcatcagctggtttagcaatagaacg (*SphI* site underlined)
- BLP2up-fw: tatgcggccgcaaaggtgacattcaggcatc (*NotI* site underlined)
- BLP2up-rv: gctggatccatcttgcagaacgtccaaac (*BamHI* site underlined)
- BLP2dw-fw: tatggatcctccagttgtttagggtatttgag (*BamHI* site underlined)
- BLP2dw-rv: tatgcatgctattggtaaacttgaactcaatgc (*SphI* site underlined)

PCR products were digested with *NotI*-*BamH*I (upstream fragments) and *BamHI*–*SphI* (downstream fragments), and cloned into *NotI*-*SphI* restricted pMo130-TelR DNA, to create pMo130-TelR-ΔBPL1 and pMo130-TelR-ΔBPL2, respectively.

Each pMo130-TelR derivative was introduced into *E. coli* S17-1 by transformation, and subsequently mobilized to the *A. baumannii* AYE strain via conjugation as described [29]. pMo130-TelR derivatives integrated into the AYE genome by homologous recombination (first crossovers) were selected on LB agar containing 30 μg/ml tellurite and 50 μg/ml ampicillin. Transconjugants were cultured in LB broth containing 10 % sucrose to select double recombinants, and serial dilutions were spread onto LB plates containing 10 % sucrose. Sucrose-resistant colonies were screened for tellurite sensitivity to monitor excision of the suicide vector. The inactivation of BLP1 and BLP2 genes in tellurite sensitive colonies was confirmed by PCR amplification, using the primers that annealed to DNA immediately flanking the deleted BLP1 and BLP2 gene regions shown below:

- BLP1mut-fw:tgtagtagatccgaaggtaatcg
- BLP1mut-rv: ccagataggtacagaagatgaagc
- BLP2mut-fw: tttggacgttctgcaagatg
- BLP2mut-rv: aattggcgcaatcctctatg

Control PCR assays were performed to rule out that merodiploids containing both the intact and the deleted gene had been selected, using the “wild-type” primers listed below:

- BLP1wt-fw: cgaaggtaatcgcaaaaattg
- BLP1wt-rv: atgtaatggacgaatgttgctc
- BLP2wt-fw: ccagatgttccacaagctca
- BLP2wt-rv: ccgcttcactggttaatggt

### Biofilm assay

Experiments were carried out in TSB (Tryptic soy broth; [31]). Overnight bacterial cultures were diluted with TSB to concentration of 10^8^ CFU/ml. Approximately 2 × 10^5^ bacteria was added to cell culture plates containing glass coverslips and incubated in static conditions at 37 °C for 48 h. The coverslips were then washed 3 times with PBS to remove non-adherent bacteria. Biofilms were stained with LIVE/DEAD^®^ BacLight^™^ Bacterial Viability kit for microscopy (Molecular Probes) as described prior [40]. The images were captured using LSM 710 inverted confocal laser-scanning microscope (Zeiss). Quantitation of mean biomass was determined using the IMARIS v7.0 software package [31]. Each experiment was performed in triplicate.

### Cell adhesion assays

Adherence of *A. baumannii* strains to A549 cells (human type 2 pneumocytes) was determined as described previously [4], with minor modifications. In brief, ~ 10^5^ A549 cells were infected with ~ 10^7^ bacterial CFU and incubated for 60 min at 37 °C in 5 % CO2 (v/v) atmosphere. Non-adherent bacterial cells were removed by washing with PBS. Infected cells were lysed by the addition of 1 ml distilled water and serial 10-fold dilutions were plated on LB agar to determine the number of CFU of adherent bacteria. To determine adherent and invading bacteria, A549 cells were infected with *A. baumannii* strains as described above. The monolayers were then treated with 1 ml of fresh culture medium containing 5 mg/L of colistin sulfate (Sigma-Aldrich, Milan, Italy) for further 30 min, the shortest time point that resulted in the killing of all extracellular bacteria added to the monolayers. Afterwards, the cells were washed with PBS, harvested with trypsin, and lysed with sterile distilled water. Dilutions from harvested samples were inoculated on LB agar plates and bacterial colony counts were estimated after overnight incubation at 37 °C. Each experiment was performed in triplicate.

### Statistical analysis

Data were analysed using Statistical Package for the Social Sciences Version 13.0 (SPSS Inc., Chicago, IL, USA). Differences between mean values were tested for significance by performing either unpaired, two-tailed Student’s t-tests or one-way ANOVA analysis followed by Tukey’s multiple-comparison test, when appropriate. A *P* value <0.05 was considered to be statistically significant. Correlations were evaluated by regression analysis using the Pearson’s correlation coefficient (r).

## Additional files

Additional file 1:
***A. baumannii*** **strains analyzed.** (XLS 84 kb) Additional file 2
**A) ABCD BAP modules in AB307-0294 BAP.** B) EGF region in type-1 and type-2 BAPs. C) Organization of type-3 BAP D) Z and Zb modules alignment. (DOC 65 kb) Additional file 3:
**BAP, BLP1 and BLP2 in non** ***-baumannii Acinetobacter*** **strains.** (XLSX 37 kb) Additional file 4:
**Organization of type-4 (A) and type-5 BAPs (B).** (DOC 42 kb) Additional file 5:
**A) NH2 and COOH regions in** ***A. baumannii*** **BAP genes.** B) solo G modules in BAPs. (DOC 52 kb) Additional file 6:
**Alpha and beta-BAP genes regions in** ***A. baumannii*** **SDF,** ***A. calcoaceticus*** **PHEA-2,** ***A. baumannii*** **AYE and** ***A. baylyi*** **ADP1 strains.** (PDF 39 kb) Additional file 7:
**A) Organization of BLP1 B) Organization of BLP2 C) BLP2 types.** (DOCX 156 kb) Additional file 8:
**BLP1 empty chromosomal site A) DNA replacing BLP1 coding sequences in BLP1-negative strains.** B) Alignment of BLP1 gene empty sites in *A. baumannii and* non*-baumannii* species. (DOCX 125 kb) Additional file 9:
**Distribution of surface proteins in strains expressing different BAP types.** (XLS 49 kb) Additional file 10:
**OmpA sequence variants.** Organization of SURP-1. (DOCX 117 kb)

## Abbreviations

aa: Aminoacids
ACB: Acinetobacter calcoaceticus-Acinetobacter baumannii
AD: Alanine and aspartic acid
BAP: Biofilm associated protein
Big_3_4: Bacterial Ig-like domain, group 3
BLP: BAP-like protein
bp: Base pair
CFU: Colony forming unit
CLSM: Confocal laser scanning microscopy
dw: Downstream
Embp: Extracellular matrix-binding protein
FIVAR: Found in various architectures
GA: G-related albumin-binding
Ig-like: Immunoglobulin-like
kb: Kilobase
KEGG: Kyoto encyclopedia of genes and genomes
LB: Luria broth
Mb: Megabase
mg: Milligram
MLST: Multilocus sequence typing
mut: Mutant
OmpA: Outer membrane protein A
ORF: Open reading frame
PBS: Phosphate saline buffer
PCR: Polymerase chain reaction
PFAM: Protein families
RADAR: Rapid automatic detection and alignment of repeats
RTX: Repeat in toxin
SD: Serine and aspartic acid
SDR: SD repeats
ST: Sequence type
SURP-1: Surface repetitive protein 1
tBLASTn: Basic local alignment sequence tool using a protein as query against nucleotide 6-frame translation
TSB: Tryptic soy broth
up: Upstream
WGS: Whole genome shotguns
wt: Wild type
XSTREAM: Variable sequence tandem repeats extraction and architecture modeling
