# Supplementary material for: Biofilm-associated proteins: news from Acinetobacter
Source: BMC Genomics. 2015 Nov 14;16:933. doi: 10.1186/s12864-015-2136-6 (PMC4647330; doi:10.1186/s12864-015-2136-6)
Supplement: Additional file 2 — A) ABCD BAP modules in AB307-0294 BAP. B) EGF region in type-1 and type-2 BAPs. C) Organization of type-3 BAP D) Z and Zb modules alignment. (DOC 65 kb) [file 12864_2015_2136_MOESM2_ESM.doc]

**S2.A** ABCD BAP modules in AB307-0294 BAP.Dashes denote identities, asterisks missing residues.

Big3_4 motifs are highlighted.

**A** (113 aa) AVGPNTDGVNFTVDSVTADNVINASEASGNVTVTGVLKNVPADAANTVVTVVINGQTYTATVDSTAGTWTVSVPGSDLTADADKTDADKTIDAKVTF**TDAAGN**SSSVNDTHTY

(111 aa) ADTTPPST---T-S--P--S--------------I------I-------A-------V--N----KA-----------G-V-*****------------**TDAAGN**-------Q--T

**B** (83 aa) DTTAPNAPVLDPINATDPVSGTAEAGSTVTVTYPDGTTATVVAGTDGSWSVPNPGNLVDGDTVTATA**TDPAGN**TSLPGTGTVS

**C** (89 aa) ADITAPVVALDDVLTNDSTPALTGTVNDPTATVVVNVDGTDYPAVNNGDGTWTLADNTLPALADGPHTITVTA**TDAAGN**VGNDTAVVTI

**D** (105 aa) DTVPADLIGAITIPEDLNGDGILNADELGTDGSFNAQVALGPDALDGTVVNVNGVNYTVTAADLANGYITAAIPVT----GEGPVAIHAEA**VDAQGN**VDVADADVTVTV

(109 aa) ------------------------------------------------------T----------------TLDA-AADPVTGQIV-----**VDAQGN**---------L-I

**S2.B** Alignment of the EFG modules of type-1 (AYE strain) and type-2 (ACICU strain) BAPs.

Identities are highlighted. Capital letters mark EFG modules as in the AB307-0294 BAP.

TYPE-1 E1 -LASSIIAFDNTDTAVLAPQPLLVQDDAALGSNTYLALVSLAGL-DLQL----GSESIGFTVGAGQEGNATFTYSALIGVDALSDYSLVVQKFDTATGQW taiygggqadil--

TYPE-2 E1 -ISSSILAFDNTDHAVLSPQPSLVGDDVSLGSTSYLVLTSVAGL-DLQL----GGNSLGFTVAAGHEGDVTFQYSGLIDAAVLSDYKLVVQKFNTTTNQW esihgdansslisl

TYPE-2 E1 -ATVTITAVDDAVNAAIAAEPHLIEDDRALGSATYLALLSLAGI-NLQAPLPFVNSTVEFNVGAGETGTATFKYSSLINEGALGDYQLVVQKFNTATNRW esitgsseasllnl

TYPE-1 E2 -QLSSVKAVDNVVTAEINPEPLLVADDVALGSSTYLAAVSLAGL-DLQL---LGNDAIEFTVDPNREGTATFTFDAVITADLLSDYAIVVQKFDEATGQW vsiggtnpeaslid

TYPE-2 E2 IVFDSIRAADNLVEVELNPQYQLVGTETDSA--FYGVLLNVGNIVDLQL---LTVDTVDFTIGAGQEGVATFNFNSLIGASALGDYNVVLQKYNDVTGQW eavngtgdrsllnl

TYPE-2 E2 GNSTPVIAVDDLAVAVVNPEYLQIGNDVAVGNTTYLALLTLTDNFDFQA----GGQSVNFTLTDATLNDVTFNYSALISASLLADYVLVVQKFDTATNQW vavngtgdadllsl

TYPE-1 F1 --DLTLLGSTPGVVIDGLEEGQYRAFMTYNGLAGIG-LLGTLTGTMDVY dttqvggyytevae

TYPE-2 F1 HLLGIGTGNVPGAVLDGLDAGQYRAFLAYDGLLGLG-VLGTLSATMDDY dlsvaggyeignae

TYPE-2 F1 SVLGIGVNATPGVVVEGLDEGQYRAFMTYNGLYGKS-ILGTLSGTMDVY dpnqidftg-lase

TYPE-1 F2 -LSLIGGTPTAVLEGLDAGQYRAFIGYEGLLGVG-LGGTLTGTMDVY npyivagysvepis

TYPE-2 F2 --TLLGNTPTAQIGGLTEGEYRAFLSFNGL-VGGAVAVTLNGSVDVY npavitgydvvaah

TYPE-2 F2 ---AAFGGNSVTLEGLAAGQYRAYMTYAGSGVGVSLLGTLSVQKDVF datnitgystqvae

TYPE-1 G1 GNVITEINDAGEVDVVTPTTVIS--EVNGQPVV---ADGTSITGTYGTLVINLDGSYTYTPTASAAGVGQTDQFTYTLTDPVTGDTAQANLN

TYPE-2 G1 GNVITDPDPTTGQVDQVTANTYV-SSVNGHPI---DADGETFAGTYGTITFYQDGSYVYVPNADGSGVGQTDVFTYTLTDSVTGATGQANLN

TYPE-2 G1 GNVITGLGVGTNADAVTGYTIVDSVTVNGVTTNVDPNTGTIIQGQYGTLQIFANGDYIYTPNNTNANLGLVDHFTYTLADPLGGNISASLDF

TYPE-1 G2 GNVIKDASLTGEVDAASSSAVIS--QVNGVAV---DPVAGATITGTGTLVIDQDGNYTYTPTVNGANLGQVDQFTYTLLDPVTG---------NTSEATLYVRLDSDSVDMTWN dadpsqp

TYPE-2 G2 GNLISDPNTNGDVDIATPNSIIS--EVNGVAV---SASPTEIIGTHGTLLIYANGDYTYTPNADSAGLGQVDQFTYTSVLGIGVNALLDPASNTTSQATFYVHLDSKVVDMNWD aadpsqp

TYPE-2 G2 GNVIHDVGLNGHADTASGFSTVTSVEFNGTAF-AVNATGVTTIVGDGTLSIYANGNYSYQPNGEAASLGQVDQFTYTLSDGL-----------NTSQATLYLHIDSDAVDMTWN tsdpsqp

TYPE-1 AVITSPLPVDAMDNVASAEIDMVYPVTTEVLDNAISYNWLLGVGGIVIGSKEGTAT--FTVDPGNLTDAIIAVNFGSVATVVDGLHVVLTRVNPDGTRTVVADSSDTGVIDLLGIFGSEVQF

TYPE-2 AVITVT-PVDAIDNVVSAGVDIVPQGELGVAVGSATYLALVGITEDLNVSLLGTPSVAFTIDAGHEADVTFAYAPVLSLSLFNDYKVVLQQKGADGAWHNIDGGSSTGLLNIGLLGNGGIGV

TYPE-1 KIDNLSAGTYELFMESNTLLTALGSVTADITLNHGDITQPPVLVVDPVTGNVLADDNSAVYGTNYVPDYITTTSV

TYPE-2 TVPDLGQGEYRAFMVYTGLGVGILGTMSVVKDDFD-YTVAPTNTAVVADGNVLTDDVTTLTTQ

TYPE-1 G3 VTAVTAENGNTTTVVVGTPATVVGVYGTLTINADGTYSYQA-TADMANVGKVDSFTYTVSDPVTGRTDTATLHVQVGSPDVDVTWN

TYPE-2 G3 VTTVTSEVVGALPQTVGTDTVINGAYGTLVISTNGHYTYTPNTTDLSAIGKVDSFTYTIRDVLTGATDTATLHVQVGSPDVTIAWD

TYPE-1 TADPSADATLPTPSVTADDDATISMAPVVDPVVDVASGNVTIGNLAGFPPLPVLSSTVTSSQFTVAANTVSDVHVQLNYTASLSL

TYPE-2 AANPANDGVVQLTANPDHVVTTTDFSNAADLPVDVASPVVSVNLIG--------SNSVVSDQFVVGAGTVANIDLSAVYTAQPLA

TYPE-1 SALPTTGYTIQQLVGTTWVDTAYSGSATALAGVL----GAPAFSADVPHLSEGTYRVVFSLSSLISLGTVTLDSVVTTTATHLDQ

TYPE-2 SVLPTVSYVIQSWNGTAWVNTIYSGSQTALGSVATIQAGSVAFQDTVEHLAAGTYRVQYTLT-GVSLGATSLDTNVSTTTVHLDT

TYPE-1 YTPDGQTDWITGNVLTND-----------VVEGTQLYVMNSTTGTYELAAGQGVNTGEGTLYLYNDGSYFYKPLDSAAN---ATV

TYPE-2 YR----SDWISGNVLAGDNIGGVADTGILSHEGAKLQVWDNAQNAYVDAVGQTINTGNGVLIMQSNGEYEYRPNDVTTTTQLAST

TYPE-1 DVIDYKLVSVIDGSEYTSSLTINLS-QELNSLAVSTAANDTFALGNGSDTLIYNTLTAASVTNATGGNTTAGGVDVWTDFHVGNT

TYPE-2 DSINYKIVSVTGGVESQSTLTIDLTHTDYNLLYTSTSANDTFTTGTGSDTVIYQLLNGTAATANNGANTGGNGVDTWTDFHVGNV

TYPE-1 ATDDQADKIDLSNLLIGSQTNLTIGQYVTVSYDAATQTATISVDRDGGLLVEGTYTETPLLQLTNLTGPVTLNDLINNGQIIF stop

TYPE-2 ATDKQADLIDIRALLDGDQTDANIGQYLNVTTSGGNTTIQIDRDGLSGLIPGNNFTTLLVLQGVTTT----ETELLNNGQILY stop

**S2.C** Type-3 BAP (AFDL, OIFC143 strain). Big3_4 motifs in modules of the

repetitive region are highlighted.

**NH2 region**

MPEIQIIAKDNHKTLVTTEGTSAKLSEASVVLVKVAASDVLVVNREGTNAVIRLKNGETIVIEGFFSGTAEPKDNSLVFQDENGQLIWAKFKDAENDADADSDA

DADADSDVEPQALLGEDLPAALPAEAPQELVSDVIYQPISSIEPLLYDDAGVNPWLWAAIPLVAGGIIAAASNHDSNDDSSTPTDT

**repetitive region**

**w** TPPATPSVPSGYLDNVGPDQGIKGSGSSTDDTTPGVVISAPGAGETPTLYVDGEKVAASYDPVTGTLTPTTPLADGSHQLTYTLTDAAGNESAQSPAITVTVDT

**w** LAPATPSVPSGYLDNVGPDQGIKGSGSSTDDTTPGVVISAPGAGETPTLYVDGEKVAASYDPVTGTLTPTTPLADGSHQLTYTLTDAAGNESAQSPAITVTVDT

**w** LAPATPSVPSGYLDNVGPDQGIKGSGSSTDDTTPGVVISAPGAGETPTLYVDGEKVAASYDPVTGTLTPTTPLADGSHQLTYTLTDAAGNESAQSPAITVTVDT

**Zb** TAPVLTISTSDLVLASGEAATITFEFSEPVSEFSVSDVVVSGGVLSNFIQVDANT--WTAIFTQSGSV-APSISVADGAYTDIVGNTGKGDVLDGADGFIFNP

**D**  LAVDLIGEITIPADINNDNILNANELGADKTFTAQITLGSDAAVGNIISVNGINYTVVQADLDAGFITAEIIVTVDGDLSVRAEARDSAGNTDIADTTIKVITVDT

**w**  TAPITPSAPTGYLDNVGPDQGIKGSGSSTDDTTPGVVISAPGAGETPTLYVDGEKVAASYDPVTGTLTPTTPLADGSHQLTYTLTDAAGNESVQSPALLLTVDT

**Zb** TAPSLVITALDPALTATESTTISFTFSEAVSGFDIDDITPVGGTLSNLVQSTTNPNVWTATFTADGSGAAPSISVADGAYTDLAGNLGTGDVLDGTDGFVVDT

**Y**  LAPAPVITIDPVTNAITIDFGEAVNAVDGSPLTADALEGLLDIANGKLTDLVDNGDGSFSGTLVPAADFEGDVVVNVPAGIVTDVAGNANLTATESLTVDT

**Zb** LAPGLVITALDPALTATESTTISFTFSEAVSGFDIDDITPVGGTLSNLVQSTTNPNVWTATFTADGSGAAPSISVADGAYTDLAGNLGTGDVLDGTDGFVVDT

**Y**  LAPAPVITIDPVTNAITIDFGEAVNAVDGSPLTADALEGLLDIANGKLTDLVDNGDGSFSGTLVPAADFEGDVVVNVPAGIVTDVAGNANLTATESLTVDT

**Zb** LAPGLVITALDPALTATESTTISFTFSEAVSGFDIDDITPVGGTLSNLVQSTTNPNVWTATFTADGSGAAPSISVADGAYTDLAGNLGTGDVLDGTDGFVVDT

**Y**  LAPAPVITIDPVTNAITIDFGEAVNAVDGSPLTADALEGLLDIANGTLTGLVDNGDGSFSGTLVPAADFEGDVVVNVPAGIVTDVAGNANLTATESLTVDT

**Zb** LAPTLVISATDTNLSLGESTTISFTFSEAVSGFDIDDITPVGGTLSNLVQSTTNPNVWTATFTQDGTLTAPSISVADDAYTDLAGNLGKGDVFDGTDGFVVNT

**COOH region**

IDAINDNVTFDPGTFVSTIWDAPIIAQDINVLDTSIIGDTSMNVEFSVPRNSADIGTGHEGDVVIQVSQDNLIAVASGFSVLVEYFDGANWVQYTTATTTNGGL

IADALGLGVLGIVDDGKTIAVHLTGVPEGQYRVVVQNDSSAIGDLLDDLTLAQLGDQGILLGADNQDAVLDAVETALNGQVLNLGTLVRNVLEPLLNIANAAGE

TLAVTDIVAAIVKIPLVGSLLGGVDTVLDYIADALVDNLLSVLEFTNVTINGSETYYTTPQVSGNVFDDNGSGADDRGNSDAALVTKVDGVDVPTDGTDAVIKG

LYGTLYINKDGVYRYVLNGSVDSNGQVDDFTYTLSDGTHSDTAVLTISVADVIAPKAPIVSSLDETTVTGVAGAAEANSVINVYGGTDSDGDGIPDTLLGTTTA

NPDGSYTVTLNPAIALGDTVVVTSKDAAGNISTPTIASLHNIVALDDPNQTNYELSTTADRPNISGGIFTLANAGVLGDLIDLDLSATSAPGVTVGTNEQITLT

LTASGSTGLDISAVGNLIDAILGGNIGGSLDLIIAKQTSTGSYEVYNTIANAYTLNNNVLTFTASGSQSISLDDAGVYKFILAPNSSTSSALSNLLSLNLLGNL

ILSSNDVVATINNTDLITGNVLSNDTAAPIGLDLSIVSIKETTAGVDETVVSGTPKVIEGMYGTLTIDSTGAYSYQMTANATALGKVEAFTYTVQDNDGHSKQA

TIYIRLDSNLVTLDWTGKTGLEEANKLVTQLTADNKASAGTVTTLFDQVLNGSGDSSNSIQVTGSNGVLPTSPPETGSTAIPDDSGRFTLGAGQTAYISFTSIL

PSNVTQDFGFLGGGNQTSAYNVVIELERDGGTLAFDTQLATWTSTTSGTKYYYKVTNTGNSSANYDLNIGYGDGSIAYNWTNTTGKTFGINNIQLDIKDTTYTA

SSVSGDLISNDILYSSVFKIGVAAGSTTPSNYLDANSTPIVGAYGTLTVNLDGTYSYQANGKIADLGKDDVFTYKIETLDGTVNTQTLTIKVDADNVLIGTPDA

GTTGNDIAVSQSANEVFTLGDGADLLIYNVLNGSSPTGGNGTDQWLDFNMSEGDKIDVSSLLSGATTDNINNYLSVSISGNQVTLLVDRDGSSGGISTPTALLT

LTNEDHATNPITSLVDLLNNNSIIY

**S2.D** Z and Zb alignment

**Z**  TAPSLVITALDPALTATESTTISFTFSEAVSGFDIDDITPVGGTLSNLVQSTTNPNVWTATFTADGSGAAPSISVADGAY**TDLAGN**LGTGDVLDGTDGFVVDT

**Zb** -TPTLAITTDDLALAAGESANITFTFSEAVAGFDANDITLVGGTLSALV—-TTDNITWTAVFTPDGTGTAPSIAVADGSY**TDLAGN**LGTGDVLDGTDGFVVDIVA
