# Supplementary material for: Biofilm-associated proteins: news from Acinetobacter
Source: BMC Genomics. 2015 Nov 14;16:933. doi: 10.1186/s12864-015-2136-6 (PMC4647330; doi:10.1186/s12864-015-2136-6)
Supplement: Additional file 4: — Organization of type-4 (A) and type-5 BAPs (B). (DOC 42 kb) [file 12864_2015_2136_MOESM4_ESM.doc]

**S4.A Organization of type-4 (A. baylyi) BAP and type-5 BAPs.**

Homologies of H repeats are highlighted

**NH2 region**

MPDIQIIAKDTHATLANITGNSAKLTQASVVLIKVPVEDVQEVTRDGTSAIVKLKNGEVIVIDDFFSTEAPTDNSLVFQDDSGKLIWAQFTDAQGALLE

NVAYQPIDSIDPLLYASNGDSSPWAWAAIPVTTGGILWWAHQHNSKDSDNQPAQP

**repetitive region**

**H** PATPTTAAIIT-DDKEPITGEVKQGGV--TNDPTPAITGSGATPGTTITIYDGDTKLGSTTVGSDGKWSFTPSTPLTDGNHDITYTVK-DTAGNESGKSPAVDFTVDTTA

**H** PATPDTAPAGT-DDQAPIVGPITSGSS--TNDNTPLISGTGTAG-EVITIYDGSTKLGTTTVGSDGKWTFTPSTPLVDGDHSIGYTAT-DAAGNESGKSPTLDFNIDTVA

**H** PTATASLVSIT-QDTGSSSSDFVTSDN--TLVFNLSTTGTLATGEYVQISLDGGTTWVNAIKGSDNSWSYDNTGKTLADGSYSIETRVVDAAGNTGTASTQVIV-VDTQS

**H** PSATAVTADDLYDDVGAIQGTISNSPDSLTDDTRPTYSGTADASVATVKVYDNGAYLGSATVGTDGKWSFTPATPIGAGSHSFTASGV-DAAGNEGPQTTAWAFKVVGAA

**H** PSAPSIQSVL--DDQGSVTGELQKNQT--TDDRTPTISGTGQVGAVVTVYVDGTAVGSS-TVAADGTWSVKTTDLGADGVKNLVAKQT-DGAGQSSPDSGAYPIVLDTTA

**H** PATPGVVTAI--DDQGVVQGPIANNGT--TDDKNPEFKGTGSAG-DVITIKDGESVLGSTTVGADGTWSFTPSTGLDEGKHSITTTAT-DPAGNTSAASDALNFTVDS--

**H** SNVVVSINKAI-DDAGSKTGDLANNAA--TDDTTPTLVGTGTVGAVVSISVDGGAVVGSAVVDSNGNWSYTLPSQSE-GTHSYTATAS-NAAGTQGTAS--FTLTIDTTA

**H** PDVPSIGQVN--DDVGLIQGPLTQGAS--TDDTTPTLTGTGATPGDVIKVYDGNDLVGSVTVGADGNWNYTIPAPGLTEGSHDLSVTATDPVGNESGKSDPFTLTVDLTA

**COOH region**

PTAPSGTFNGGGSEITGSAEAGSVVKVKDANGNVLGSATADSSGKYTVTLADPLNDAETVKITATDKAGNESQATDLTAPNVIIDAEDNIVEAKVDFTYPVTVNPSETIINE

TSLINIGTSTYPGYFTVGQDQIADAVISVSTGSLINLFDNAEMKLYKQQDDGSWELIADNQSPGLLDLLGIFGQTTKVTAEGLTPGNYRFDFTGGSLIGLGTSIKADLQLTT

QNTAANPVVGDITSKEGNVITDADATNGQDQVTSQTKVTAVNGQTVAADGTTTIVGEHGTLTIKADGSYKYTPNSDVTVIGKTDTFNYTITDASTGKSDTAKLIIQIGTNSD

LDLTWNPNNPEADATSVVATNDEDTVGIGATNTETTTAGPSINESWLIGLGGSQTVTSQTITIAQGNLGAVEIGLSTSSLLGLGGAASVQFQKLVNNTWTTVDTISASSLAD

LIGLFPNGTGKVYDDLEAGQYRYILNYNRGLGVAGNVSVSSEVTSTDLDSYTITSRETVQGNVLTDDTGAGVDKVASHYTDVAISHDGLTYTTVTSTGISILGTHGTLVIKS

DGSYTYTPNSTLTDGGEDQFTYKLIAPNGDESTATLTFNAGFVYNTSAGADIITSSAGNDTYTTHGGADTVIFKLLNSTDATGGNGHDTWTDFSKADGDKIDITALLSGQSV

SSSTINNYVTVTTKGADTVISIDRDGSAGHTYDSTELLTLKNVNTTLDELLQHNQLLF

**S4.B Organization of type-5 (A. radioresistens) BAP.**

Stretches of aminoacids inserted between repeats are in red.

**NH2 region**

MADISIISKETAQQVEHIGNSFKLGEPVVVVIDVAPEEVASIDRSGNNLTITLKNGEQIYIENYFAADNSLVFKNDQNQLLLAQVTDASGAILDPISYLNLEEVTPLLYGAE

SEAFVPWLVGAVGIGGLAAAVTSTSDSSDDTRN

**repetitive region**

**B** DITPPQKPIITENNGDGLSGTGEAGSTVIVKDSQGNQTEIKVGEDGKWSIEPNPLNEG-EQGTIEAVDPAGNSSGQSPV

**C** TGGDQTVPQATLDTLLTNDTTPGLSGTVNDPAARIEVSINGSTYQAINNGDGSWSLADNIIAVLPEGLINVTVTATDAAGNRSAVTGSIRID

**B** TTAPAAPLLDAVNATDPISGTAEAGARIVVTYPNGTTASTVADAAGNWSVANPGNLSDGQQISVVASDAAGNSSAPASA

**C** TVDADITAPEVSLDALLSNDTTPGLSGTVNDPAARIEVSINGSTYQAINNGDGSWSLADNIIAVLPEGLINVTVTATDAAGN RSAVTSIRIDG

**B** TTAPAAPLLDAVNATDPISGTAEAGXRIVVTYPNGTTASTXADAXGNWSVANPGNLSDGQXISVVASDAAGNSSAPASA TVDADITAPSLE

**M** FIVIINADGSATLSGTTEPGTSVIVQGPDGAAVPVNVAPDGSISGSIAAPALAGSYSATATDINGNTATDTATATDSTPPAVE

**M** LNVVINTDGSATLSGTTEPGASVTVQGPDGAAVPVTVAPDGSISGSIAAPALAGNYTATATDINGNTATDTATAIDNIAPIPGSL

**N** SLADFSDTGISLSDGISNDNTFNLTLTGQETGSTVVYQISADGGNTWVATTAAQTGVADNTYQYRAVVTDVNGNSSVTDTVNVTVD

**N** SLADFSDTGISLSDGISNDNTFNLTLTGQETGSTVVYQISADGGNTWVATTAAQTGVADNTYQYRAVVTDVNGNSSVTDTVNVTVD

**B** TAAPDAPELDPVNVTDPISGTAEIGATVTVTFPDGTIATALADESGSWSVANPGGLANGDIITAIATDPAGNASLSGSG

**C** TVSADITAPVVVLDDSSSRDNTPPLSGTVNDTTARIVVTLNGTDYEAVNNGDGTWTLADNTLPVLQDGTYNLTVRAIDQAGN TGTDTATLTIDTITTVE

**M** LNVVINADGSATLSGTTEPGASVTVRGPDGAAVPVTVTPDGSISGSIAAPALTGNYTATATDING-TATDTATATDSTPPAVEN

**M** LNVVINADGSATLSGTTEPGASVTVRGPDGAAVPVTVTPDGSISGSIAAPALAGNYTATATDINGNIATDTATAIDNIAPIPGSL

**N** SLADFSDTGISLSDGISNDNTFNLTLTGQETGSTVVYQISADGGNTWVATTAAQTGVADNTYQYRAVVTDVNGNSSITNAVGVTID

**COOH region**

TITTVTARFALDSDNGNPDPTTVIVSGTAEVGSTVTLTTTTGVAILDNLGNPVSTIVGSTGTYTFNVPTASVADNQLLSVNSTDIAGNVATDPVRIDLTPPNAIDDVVNLD

IVSQITTVYPEVTDTDVQVLGLLESDNGTDNGVTVIVGPDQFGVLNIEISQTALAAVADAFRLDVVDANGNVVYSAVTQNSLLGDVLGLNVLGVTGDNTLTATVRGLQPGTY

RVVVRNDESTLTDLLDSDNSGGVSLQELGNSGVILGPENQTLVLNTVSNALGPVLGPTVTTLLSPVLTTLNGLPVDEIINPLVTILNNVGATGLLNTVLSSLADALLSNTLT

LLQATDITTQLTETDFANEALTGGNVITGAGSSNGGTDDAQGGVINQVQIADGPIVVIPSNGSNITVEGLYGVLTINSSGAYTYTAYGDPASIGGSEVFTYTLSDGNTVDTA

KLTINIADTSTTAPRVTLVNDTGNSNTDLITQDGRIAVTADPDVNTVVVRYTSASGTGVNVNAVYDTATGRWIATPPAGFADGTYNVTATVTDTTNHANTSSLGNITLDTTP

PVVTIVGAGDNVGNIQGNIYSGAITDDNTPTIFGRAAPNSTITLIQDGVTITTPITVDASGNWSYTPATALSSSATGVNHTWQARVTDTAGNITTANFNLNIIGQVAPVAAV

NSNALLGVVGADVAGLIDLNQQLFAAADANGDLRRVEITLNSAVSLGGENFSFSTGLRNLFGYNVTTQSSTVLGRPARITIEAAQGQVFDNQEINEFLASVKLSGGLLGGIL

NLNLLNNLNVVATDSQGSSTSYSRGEIASVDLLGGLLSGSASPIVEGNANANILGNSNSTVSQRLYGYDGNDTLNGGSGNDILRGGNGDDTLNAGAGNDYINGGAGNDTITG

GSGADTVVFDLLVAANATGGNGTDTWTDFHVGNIRTDNQADRIDVSDLLGGEVNANNLGQYIQLNYNSTSSTVTLSIDRDGTGTTFIATPLLQLTNQPSAITLDELLQNGQI

IF
