# Supplementary material for: Biofilm-associated proteins: news from Acinetobacter
Source: BMC Genomics. 2015 Nov 14;16:933. doi: 10.1186/s12864-015-2136-6 (PMC4647330; doi:10.1186/s12864-015-2136-6)
Supplement: Additional file 5: — A) NH2 and COOH regions in A. baumannii BAP genes. B) solo G modules in BAPs. (DOC 52 kb) [file 12864_2015_2136_MOESM5_ESM.doc]

**S5.A Homologies at the NH2 (A) and COOH (B) terminus of different BAPs.**

*A. calcoaceticus* 1 and 2 refer to the PHEA-2 and AIEC genomes, respectively.

BAPs in AIEC and all other *A. calcoaceticus* draft genomes are similar.

A)

*A. baumannii*  all types MPEIQIIAKDNHKTLVTTEGTSAKLSEAS--VVLVKVAASDVLVVNREGTNAVIRLKNGETIVIEGFF-SGTAEPKDNSLVFQDENGQLIWAKFKDAENDA

*A. baylyi*  ty-4 MPDIQIIAKDTHATLANITGNSAKLTQAS--VVLIKVPVEDVQEVTRDGTSAIVKLKNGEVIVIDDFF—-STEAPTDNSLVFQDDSGKLIWAQFTDAQGAL

*A. radioresistens*  ty-5 MADIFIISKETAQQVEHI-GNSFKLGEPVVVVIDVAPEEVASIDRSGNSLT--ITLKNGEQIYIENYF------AADNSLVFKNDQNQLLLAQVTDASGAI

*A. baumanni SDF*  ty-6 MSEIRVISKDSHETLEITTKDTVSLSEAS—-VILIKVNKDDVSEIRQDGRNAIITLKNGEQIVIVDFF-NGSNYSTDNSLVFEDNNHKLIWVQFTDANGAL

*A. calcoaceticus 1* ty-6 MSEIRVVSKESHETLEITTKDTVSLSEAS—-VILIKVNKDDVSEIRQDGRNAIITLKNGEQIVIVDFF-NGSNYSTDNSLVFEDNNHKLIWVQFTDANGAL

*A. pittii*  ty-6 MSEIRVVSKESHETLEITTKDTVSLSEAS—-VILIKVNKDDVFEIRQDGRNAIITLKNGEQIVIVDFF-NGSNYSTDNSLVFEDNNHKLIWVQFTDANGAL

*A. calcoaceticus 2* ty-7 MSEIRVISKESHETLEITTKDTVSLSEAS--VILIKVNKDDVSEIRQDGRNAIVTLKNGEQIVIVDFF-NGSNYSTDNSLVFEDNNHKLIWVQFTDANGAL

*A. haemolyticus*  ty-8 MSEIQIISKESHKTLLNTTDNTTALSASQPAVVLIKVPIEDVAQVKRDGTNAVVFLKNGEQIVIQNFFANDNNQNLDNSLVFQGQDGKLIWARFKDSESDS

*A. baumannii*  all types DADADADADADADVEP--QALLGEDLPAALPAEAPQELVSDVIYQPISSIEPLLYHDA-GVNPWLWAAIPLVAGGIIAAASNHDSNDDSSAP

*A. baylyi*  ty-4 ------------------------------------ALLENVAYQPIDSIDPLLYASNGDSSPWAWAAIPVTTGGILWWAHQHNSKDSDNQP

*A. radioresistens*  ty-5 --------------------------------------LDPISYLNLEEVTPLLYGAE-SEAFVPWLVGAVGIGGLAAAVTSTSDSSDDTRN

*A. baumanni SDF*  ty-6 --------------------------------------LENITYSYIDSIEPLLYHDG-VASPWAWLSVPLTAAGILWWAHDSDDKNNNLNS

*A. calcoaceticus 1*  ty-6 --------------------------------------LENITYSYIDSIEPLLYHDG-VASPWAWLSCSK

*A. pittii*  ty-6 --------------------------------------LENITYSYIDSIEPLLYHDG-VASPWAWLSVPLTAAGILWWAHDSDDKNNNLNN

*A. calcoaceticus 2*  ty-7 --------------------------------------LENITYSYIDSIEPLLYHDG-AASPWAWLSVPLTAAGILWWAHDSDKNNSLES

*A. haemolyticus*  ty-8 DADADADADADADIEPDMEAAVYLEETPVAIPPVVQAPVSDAIFQPIDSITPLLYHDG-GISPWLW-GVPLVAGGIIAAASGGGSKNRNDESDS

B)

*A. baumannii*  ty-1 AGG--VDVWTDFHVGNTATDD--QADKIDLSNLLIGSQTNLT-IGQYVTVSYDAATQTA--TISVDRDGGLLVEGTYTETPLLQLTNLTGPVT--LNDLINNGQIIF

*A. baumannii*  ty-2 TGGNGVDTWTDFHVGNVATDK--QADLIDIRALLDGDQTDA-NIGQYLNVTTSGGN-TT---IQIDRDGLSGLIPGNNFTTLLVLQGVTTT----ETELLNNGQILY

*A. baumannii*  ty-3 TGGNGTDQWLDFNMS--------EGDKIDVSSLLSGATTD--NINNYLSVSISGNQVT----LLVDRDGSSGGISTPTALLTLTNEDHATNPITSLVDLLNNNSIIY

*A. baylyi*  ty-4 TGGNGHDTWTDFSKA--------DGDKIDITALLSGQSVSSSTINNYVTVTTKGAD-TV---ISIDRDGSAGHTYDSTELLTLKNVNTT------LDELLQHNQLLF

*A. radioresistens*  ty-5 TGGNGTDTWTDFHVGNIRTDN--QADRIDVSDLLGGEVNAN-NLGQYIQLNYNSTSSTV--TLSIDRDG-TGTTFIATPLLQLTNQPSAIT----LDELLQNGQIIF

*A. baumanni SDF*  ty-6 TGGNGKDEWTDFNLA--------QGDKVDISSLLNG—-ANASNISNYVSVTSDGAGNTL---ISIDRDG-TGNTYNSTDLIVLKNTDTT------LDELLNNNQLLF

*A. calcoaceticus 1* ty-6 TGGNGKDEWTDFNLA--------QGDKVDISSLLNG--ANASNISNYVTVTSDGAGNTL---ISIDRDG-TGNTYNSTDLIVLKNTDTT------LDELLNNNQLLF

*A. pittii*  ty-6 TGGNGKDEWTDFNLA--------QGDKVDISSLLNG--ANASNISNYVSVTSDGAGNTL---ISIDRDG-TGNTYNSTDLIVLKNTDTT------LDELLNNNQLLF

*A. calcoaceticus 2* ty-7 TGGNGSDTWSDFNKA--------EGDKIDISTLLSGQSVNNTNIGQYVTATQVGAD-TV---ISIDRDG-SGISYNSTEILTLKNTTTT------IDELLQNSQILF

*A. haemolyticus*  ty-8 TGGNGHDHDTWFNFHVGDTAIDSEADKIDISDLLVGYAGDGSAASLSAYVSVDFDGTDT--IISIDRDGGAGVHS-STELLTLKNVDTSL-----EELLQNN-QLLF

**S5.B Solo G modules in different BAPs**

A. baumannii ty-1 VTAVTAENGNTTTVVVGTPATVV GVYGTLTINADGTYSYQATADMAN-VGKVDSFTYTVSDPVTGRT DTATL-HVQVGSPDVDVTWN

A. baumannii ty-2 VTTVTSEVVGALPQTVGTDTVIN GAYGTLVISTNGHYTYTPNTTDLSAIGKVDSFTYTIRDVLTGAT-DTATL-HVQVGSPDVTIAWD

A. baumannii ty-3 VSIKETTAGVDETVVSGTPKVIE GMYGTLTIDSTGAYSYQMTANATA-LGKVEAFTYTVQDN-DGHS-KQATI-YIRLDSNLVTLDWT

A. baumannii SDF ty-6 SIANENGVVIALPQDQNGSTRIQ GEYGELFIDKNGNYEYIRDFTIPNSLGKVDSFTYTIQDS-DGHQ-DTATL-NVRIDTNDLDITWP

A. pittii,APQH ty-6 SVANEDGVITALPQDSEAFTRIQ GEYGELFIDKNGNYEYIRDFTIPNSLGKVDSFTYTIQDS-DGHQ-DTATL-NVRIDTNDLDITWP

A. calcoaceticus 1 ty-6 SVANEDGVTTALPQDSEAFTRIQ GEYGELFIDKNGNYEYIRDFTIPNSLGKVDSFTYTIQDS-DGHQ-DTATL-NVRIDTNDLDITWP

A. calcoaceticus 2 ty-7 QVSSETGNTLTVDQSVNGFQKIE GAYGDLYIDKDGNYEYIRNPEIPNSVGKVDTFTYTIQDV-DGNT-ATANL-NIRIDSSQVDINWP

A. baylyi ty-4 SQTKVTAVNGQTVAADGT-TTIV GEHGTLTIKADGSYKYTPNS-DVTVIGKTDTFNYTITDASTGKS-DTAKLIIQIGTNSDLDLTWN
