# Supplementary material for: Biofilm-associated proteins: news from Acinetobacter
Source: BMC Genomics. 2015 Nov 14;16:933. doi: 10.1186/s12864-015-2136-6 (PMC4647330; doi:10.1186/s12864-015-2136-6)
Supplement: Additional file 6: — Alpha and beta-BAP genes regions in A. baumannii SDF, A. calcoaceticus PHEA-2, A. baumannii AYE and A. baylyi ADP1 strains. (PDF 39 kb) [file 12864_2015_2136_MOESM6_ESM.pdf]

ADDITIONAL FILE 6 α-BAP and β-BAP genes in A. baumannii SDF and AYE, A. calcoaceticus PHEA-2, and A. baylyi ADP1 genomes. Homologous ORFs are in blu, transposase and resolvase genes are highlighted

A. baumannii SDF		A. calcoaceticus PHEA-2	A. baumannii AYE	A. baylyi ADP1
ABSDF0775	2-oxoglutarate dehydrogenase E1 component	BDGL_002149	ABAYE0780	ACIAD2876
ABSDF0776	2-oxoglutarate dehydrogenase E2 component	BDGL_002148	ABAYE0781	ACIAD2875
ABSDF0777	dihydrolipoamide dehydrogenase	BDGL_002147	ABAYE0782	ACIAD2874
ABSDF0778	succinyl-CoA synthetase subunit beta	BDGL_002146	ABAYE0783	ACIAD2873
ABSDF0779	succinyl-CoA synthetase subunit alpha	BDGL_002145	ABAYE0784	ACIAD2872
-	hypothetical protein	-	ABAYE0786	-
ABSDF0782	tryptophanyl-tRNA synthetase II	BDGL_002144	ABAYE0788	ACIAD2871
ABSDF0784 tpase	hypothetical protein	BDGL_002143	ABAYE0789	ACIAD2870
-	hypothetical protein	BDGL_002142	ABAYE0790	ACIAD2869
-	Na+/H+ antiporter	BDGL_002141	ABAYE0791	ACIAD2867
ABSDF0785	**α– BAP**	BDGL_002140	ABAYE0792	ACIAD2866
-	hypothetical protein	BDGL_002139	ABAYE0793	-
-	bifunctional poly-gamma-glutamate biosynthesis	BDGL_002138	ABAYE0794	-
-	metalloprotease	BDGL_002137	ABAYE0795	ACIAD2865
-	methyltransferase	BDGL_002136	ABAYE0796	ACIAD2864
-	universal stress protein A (UspA)	BDGL_002134	ABAYE0797	ACIAD2863
-	chloramphenicol acetyltransferase	BDGL_002133	ABAYE0798	-
ABSDF0788	transcription elongation factor GreA	BDGL_002130	ABAYE0799	ACIAD2862
ABSDF0789	carbamoyl-phosphate synthase large subunit	BDGL_002129	ABAYE0800	ACIAD2861
ABSDF0790	carbamoyl phosphate synthase small subunit	BDGL_002128	ABAYE0801	ACIAD2860

	ABSDF2728	5-methyltetrahydropteroyltriglutamate	BDGL_003590	
	ABSDF2727	flavoprotein oxidoreductase	BDGL_003591	
	ABSDF2726	pseudogene	-	
	ABSDF2725	pseudogene	-	
	ABSDF2724 tpase	transposase		
	ABSDF2723	ribonucleoside-diphosphate reductase subunit beta	BDGL_000011	
	ABSDF2721	ribonucleoside-diphosphate reductase subunit alpèha	BDGL_000013	
	ABSDF2319 tpase			
	ABSDF2318	lap E type I secretion outer membrane protein	BDGL_003595, 3596	
	ABSDF2317	lap B secretion efflux system (ATP-bind and membrane)	BDGL_003597	
	ABSDF2316	lap C secretion efflux system, membrane fusion protein	BDGL_003598	
**GEI**	ABSDF2314	**β–BAP**	BDGL_003599,00001	
	-	hypothetical protein	BDGL_000002	
	ABSDF2312	glycosyltransferase	BDGL_000003	
	ABSDF2311	putative acetyltransferase, cysElacA/LpxA/NodL family protein	BDGL_000004	**GEI**
	ABSDF2310	hypothetical protein	BDGL_000005	
	ABSDF2309 tpase	hypothetical protein	BDGL_000006	
	-	hypothetical protein	BDGL_000007	
	-	hypothetical protein	BDGL_000008	
	ABSDF2308	resolvase	BDGL_000009	
		resolvase	BDGL_000010	
