# Supplementary material for: Biofilm-associated proteins: news from Acinetobacter
Source: BMC Genomics. 2015 Nov 14;16:933. doi: 10.1186/s12864-015-2136-6 (PMC4647330; doi:10.1186/s12864-015-2136-6)
Supplement: Additional file 7: — A) Organization of BLP1 B) Organization of BLP2 C) BLP2 types. (DOCX 156 kb) [file 12864_2015_2136_MOESM7_ESM.docx]

**S7.A Organization of BLP1**

The sequence of the AYE ORF821 is shown. Changes in the COOH region among type variants are highlighted.

**NH2 region**

MKFHGGKMVQVSLMANNASKNNTVDINIGQTKNIVVDPKVIAKIDINPEKIASITRDGNSAVIHLKDGTEIVLENFFISENPQI

LLNEGQTYWAANLAEDATGQTTVNYLELKEIPKYIDASSSVPIWSWVVSALAGAGTVALLSQQDAK

**Repetitive region**

**P** DKIPPEPGKLSFQNLLDSGELTQDQITNDNKFNLKLSGQEKGSSVTYLIST-----DEGKTWQETTLNQKDLADGIYLYKAVVTDAAGNTSETAVQKVVV

**P** DTTAPQAGELTLSDLSDTGISATDQITQDKNFNLKLEGQESGSRVTYLVST-----DEGKTWQETTIAQKDLTDGVYQYKAVVTDAAGNTSETAVQKVVV

**P** DTTTPQAGELTLSDLNDTGVSVTDQITQDKNFNLKLEGQETGSRVTYLVST-----DEGKTWQETTVVQKDLTDGVYQYKAVVTDAAGNTSETAVQKVVV

**P** DTTTPQAGELTLSDLNDTGVSVTDQITQDKNFNLKLEGQETGSRVTYLVST-----DEGKTWQETTVVQKDLTDGVYQYKAVVTDAAGNTSETVVQKVVV

**P** DTTTPQAGELTLSDLNDTGVSVTDQITQDKNFNLKLEGQETGSRVTYLVST-----DEGKTWQETTIAQKDLADGVYKYKAVVTDAAGNTSETAVQKVVV

**P** DTTAPQAGKLTLSDLNDTGVSATDQITQDNSFTLKLAQPIVIGEQAALLDHYEVSKDEGKTWQETTADQKDLADGIYQYKAIVTD-LGNISESAIQKVVV

ADNSLNVESTTVIVKPITEDNTISLVEKDQVISIRLEIANLPTDLNSSLTSVNTTLGNVTYNFHFDEVTQEWVTEIPAEFLWSVEPQTNISIEISLTDQ

AGNTAIIKHTQNYNVDHTPNSPTLDSLTFNNIDGAIISGSAYKGSKVDIYNKNGDWLASTITNEEGKFTLQDLSINSNQEVYAVATYNGYSSENSSIGL

VTEVPAISITRISPEGVISGYATEGSHFIVKDQNGNILQEFNSNVFDSSGITPFSVMALGEVRPFILSLDQPLEEGAQIIISIDKDNISGHPQYITADY

**Q** TPAVFLETPQFDISGETLSVHVNEPNSFIRAFSGEGNLIATGF----TDEQGFASLQVFQFLKEGETVSVQVVDKNQNTSETLIE-VPNFAYIPHVERITQEGLISGVAEDNSTVIVRDADGNELGKVTLGDDNSWSDFSHFSLSVNRPLIDGEKISVQIIDNKGLMSPEQNIIVDL

**Q** TPPPAPTELNF-NDAGDLVYGHAEPFSEILVKDGQGNILNKWFWNNWTDESGSFSIELGTFLTNAETVYVTATDVNGNVSLAAQIQAPNYAFAPYVDSFTSDGVISGQAENNSTLVVKDAKGDVVAEIKVGEDNGWNGSSYFKLQLDRPLVDGEQFFLSIKDARGQVSADTVITADT

**R**  VAPTPSANLVFSEDGSYLTGVAELNTTIQVFDHNGQLVNI WNNTINSDGTFTIYLGSNNLHGEAFTVTVKDQAGNVSEAISINAPLDD

**S**  IAPNPIKNILLDANGQNFTAQAEANSQIEVFDSLGNQTGW GSTDSAGNVSGSFNQTY--LHGEELTFVVIDRAGNRSIEFKQNALIDT

**S**  IAPNPIANIIFNEDGQSFTAQAEAGSSIDVLDQTGNKIGF GYTDSSGNVSGYFQQVY--LHGEELTFVVIDRAGNRSAEVKQSALNDD

**S**  VVPNPIENIVLDLNGQNFTAQAEANSQIEIKNNNGDVVGY GSADSAGNVSGYLYQVH--LHGEELTFIVVDRAGNRSTEVKQNALIDD

**S**  IAPNPIENIVLDINGQNFTAQAEANTQIEVKNAVGEIVGL GYVDGAGNVSGYLYQVY--LHGEELTFVVVDRAGNRSTEVKQNALIDD

**S**  IAPNPIENIVLDINGQNFTAQAEANTQIEVKNAVGEIVGL GYVDGAGNVSGYLYQVY--LHGEELTFVVVDRAGNRSTEVKQNALIDD

**S**  IAPNPIENILLDANGQNFTAQAEANTQIEVKNTAGEVIGS GSTDSMGNVSGYFYQVY--LHGEELTFVVVDRAGNRSTEVKQNALIDD

**S**  IAPNAIENIIFNENGQNFTAQAEANSKVEVKNAAGEVVGS GYVDSVGNVSGYLNQVY--LKGEELTFVVIDQAGNRSIEVKQTAFLDN

**R**  TAPENATNLVFSEDGSYLSGMAEPNATIQIFDQYGQLLNQ WNNNVNWDGTFNIYLNSNYMHGEVFKVVVVDHAGNLSGEVTVKAPLDD

**R**  IAPVAASDLVFNEDGSSLSGVAEPNTFIQIFDQNGQQMNT WSQSVNADGTFTIFFGTYNLHGEEFTVIVKDLAGNVSEAVSVKAPLDD

**S**  IAPKPIKNIVFDANGQSFTAQAEANSQIEIFDSFGSQIGW GSTDSTGSVTGYFYQVY--LHGEELTFVVIDRVGNRSDEMKLNALMDT

**S**  IAPKPIENIIFNENGQNFTAQAEANSFISVKNAAGEFVGY GYVDSTGNVSGHFNQVY--LKGEELTFIVIDKAGNQSIEYKQNALTDD

**S**  IAPNPIENIVLNKNGQNFTAQAEADSQIEVKNTAGEVVGS GYVDSIGNVSGSFNQVY--LHGEELTFVVVDRAGNRSTEVKQNALIDD

**S**  IAPNQIENIVFDVNGQYFTGHAEADTRIEVLDQFGNRAGW GYVDSQGNVIGYFNQVY--LHGEELTFIVVDIAGNRSVEVKQNALIDN

**R**  VAPPAAANITLTSDGL-LFGEAEPNSTVEIIDQYGAVITT TYV--WYDGTFNQWINLSQYQTQNLSIVVKDQAGNRSEVVHELVPVFT

**R**  NSPIAATELKLDIDGHILTGKATVGMSVVVTSTDGQTINGGWNNAVNEDGSFAIQLNDYYLQGQTLQVRVYDQNTNQYSLISEIIAPLD

**COOH region**

type 1 NIAPVINEVVINNDGYGITGQTDSKAIIQVMDADGDLRAEFQADETGYFNASIYPPILRGEQLFITAIDLAKNISKPFNITFNADTNAPPSAEHIVVSENGFFIEGTAVAISTVHIFDVHS

type 2 NIAPIINDVVINNDGYSITGHTDPKVIIKVMDEDGDFRAEFQSDEAGYFNASIYPPLLRGEQLFITATDLAKNISTPFNITFNADINAPPSANHVVISENGFFIEGTALPNSVVRIYDVYS

type 2H NIAPIINDVVINNDGYSITGHTDPKVIIKVMDEDGDFRAEFQSDEAGYFNASIYPPLLRGEQLFITATDLAKNISTPFNITFNADINAPPSANHVVVSENGFFIEGTALPNSVVRIYDVYS

type 2K NIAPVINEVVINNDGYGITGQTDSKAIIQVMDADGDLRAEFQADETGYFNASIYPPILRGEQLFITAIDLAKNISKPFNITFNADTNAPPSADHVVLSENGFFIEGTAVAISTVHIFDVHS

type 1 NHVATNVADEAGNFNIQLYPPLASGQILRIVVEYNGYQSAYTEITAPIDTVAPNAATQLLLEDGNVLSGQAEAYSIVNIFDANNNLVGQTNVGSDGAFLTHLWYEYWHGETLTVKVVDANQ

type 2 NYIGGGFVDETEHFNIQLYSPQANGQTLRVVVEQNGYQSAYTEITAPIDTVAPNAATQLVLEDGNVLSGQAEAYSTVNIFDANNNLVGQTTVGSEGSFLTQLWSQYWHGETLTVKVVDANQ

type 2H NYIGGGFVDETEHFNIQLYSPQANGQTLRVVVEQNGYQSAYTEIKAPIDTVAPNAATQLVLEDGNVLSGQAEAYSTVNIFDANNNLVGQTTVGSEGSFLTQLWSQYWHGETLTVKVVDANQ

type 2K NHVATNVADEAGNFNIQLYPPLASGQILRIVVEYNGYQSAYTEITAPIDTVAPNAATQLLLEDGNVLSGQAEAYSIVNIFDANNNLVGQTNVGSDGAFLTHLWYEYWHGETLTVKVVDANQ

type 1 NVSVGTTIVAINDTVVPDVVTQLAIDEWGS-LTGRVESYATVELTYHFTDQPLSVTSTTALANGMFFIYLDRNATSLDLTVIDRAGNRSETISQIISDLPTVIIDHFKGDATDNTYNIDTI

type 2 NVSVSTTITATNDTTAPKVVTQLAINEWGNWLVGHAESNATLEITYYFADQEPSVTSTTVMADGTFSTYVYGTATSFDLTVIDRAGNRSETISKAINDLPTITVDQFKGDATDNTYIVDHI

type 2H NVSVGTTITATNDTTAPKVVTQLAINGWGE-LTGHAEGKATLEITYYFADQEPSVTSTTVMADGTFSTYVYGTATSFDLTVIDRAGNRSETINKAINDLPTITVDQFKGDATDNTYIVDHI

type 2K NVSVGTTIVATNDTTAPKVVTQLAINEWGNWLVGHAESNATIEVTYYFADQEPSVMSTTVMADGTFSTYMYGNATSFDFTVIDRAGNRSETISKAINDLTTITVDQFKGDATDNTYVVDHI

type 1 DDFVQEYIV-------EPYAIYKDVWIDNSYMYSDWVIEGHYEQIWFVDGYYE----------SQWA-SGYSTVQNIYQNQNGITYIDNGTADSDYSRYEQQYYDFVNGQWQEGYELTYIR

type 2 SDFVEEYTV-------EPYAIYKDVWIDNSYMYPEWVSEGHYEQIWFVDGYYD----------SQWITSGYSTVQNVYQDQNGITYIDNGTADSDYSRYEQQYYDFVNGQWQEGYELTYIR

type 2H SDFVQEYTV-------EPYAIYEEIWIDNSYMYPEWINEGHYEQEWLVSGYYEQKLITNAFYETQWIPNVHEVVLNIYQTQYGMAYIDNGTPESEYSQYVQQYYDAVTGQWQVGYELTYVR

type 2K SDFVQEYGVALVEVFEDVWIDTSHYEEQWVASGYTDYIWVDTSHFENVWIDTSYSQDIWIDTSYYQDVWIVDGTRDVYTDQNGVNY---YSDDGSYNQYVNSYYDYNLNQWQSGYGLTGPY

type 1 SEEGWVDTSHYEDVYI ---------------DTSHYEEVWVDTSHYQDIWVENSYWESQLVESGRRDVDLGGHDKIISSVNYSLVGYQTVNDPTTVDSFL--LESGRYVEDLELVGSAHL

type 2 SEEGWVDTSHNEDVYI ---------------DTSHYEDVWVDTSHYEDVWVESGYWENQLVESGYRDFDFGGHDKIVSSVSYSLVGSDWSTG----------LESGRYVEDLELVGSAHL

type 2H SQMGWVDTGHYEDVWVETSYYEDVYI------DTSHYEDIYVDTSHYEDVWVESGYWESQLVESGSRDVDSGGHDKIISSVSFSLVGNSQWVFNNETSGSILIIESGRYVEDLELVGSAHL

type 2K TEEIGHFEQQFVQDGHYESQWIEQGYYEQQYVFDGHNEETWVDTSHYESIWVQNGYWEPQLVGFEYKDVDLGGHDKIISSVNYSLVGYQTVNDPTTVDSFL--LESGRYVEDLELVGSAHL

type 1 NATGNALDNLLTGNSGNNVLNGREGNDTYITNEGTDTIVFQLLNSQDATGGNGHDTVLDFTLGDIRTNLQADKIDLSELLIDYSKDVSALAKFITVEQDAGNTTISLDRDGEGTMFNSVSL

type 2 NATGNALDNLLTGNSGNNILNGREGNDTYITNEGTDTILFQLLNSQDATGGNGHDTVLDFTLGDIRTNLQADKIDLSELLIDYSKDVSALAKFITVEQDAGNTTISLDRDGEGTMFNSVSL

type 2H NATGNALDNLLTGNSGNNVLNGREGNDTYI-NEGTDTIVFQLLNSQDATGGNGHDTVLDFTLGDIRTNLQADKIDLSELLIDYSKDVSALAKFITVEQDAGNTTISLDRDGEGTMFNSVSL

type 2K NATGNALDNLLTGNSGNNVLNGREGNDTYI-NEGTDTIVFQLLNSQDATGGNGHDTVLDFTLGDIRTNLQADKIDLSELLIDYSKDVSALAKFITVEQDAGNTTISLDRDGEGTMFNRVSL

type 1 LTLNQVNTTLDELLNNQQIIV

type 2 LTLNQVNTTLDELLNNQQIIV

type 2H ITLNQVNTTLDELLNNQQIIV

type 2K LTLNQVNTTLDELLNNQQFIV

**S7.B** Organization of BLP2

The sequence of the AYE ORF1037 is shown.

**NH2 region**

MTRIIVASKEGLDVLQDGQLNKVVLNQPTIIQIGVSQKDIASMEKQGGSLVIHLKNGETIVLENFFNEATNTTEHSLVFPTEQGKFVEAQF

DAQGKVIDYRGLNHVTDLAYTSTSPSAATMAVDNDPSFSMGNVLKAGLAVLAAEGLYLWAFDKDDKDDSPSTPDLIAPAAPTATLADDTV

**Repetitive region**

**T** TVTGKTEANAKIYIKDAAGNTVASGVADASGNYTIKLDKPLVNGDKLNVIAQDAAGNNSKVTVVTGTKDTIAPDVPQAQLSDDGS

**T** LLTGKAEANAKITVYDATGKVLGTVFANKDGIYSLKLTPPLTSEAGGKVVAEDAAGNKSEEVKIIAGKDTIPPASPFVEVNKEGS

**T** VIHGKTEANAKVQIKDADGKVIGSGTADAQGEFQITLSPALKEAQKGTVVVEDAAGNVSKPVEITPGFDSIAPDKPTVQINTDGT

**T** SVTGTAEANAKIEIKDTTGKVIGSGTADANGKFTISISPALTDNKHASVSAIDNAGNKSEVVDIVGTKDTTPPAKPILNSVDDDV

**COOH region**

GAVKGAITAGSETDDARPKLTGSGEANATLTIYDNGVAIGVVTVTSGRSWSFTFDKDLALGKHTITLTQTDAAGLTSEASSPFTFYVVAPKAAS

LSETSVDILSTEGPSLADSVGLHTLKVAQNTTTETNNPQKSVPLDDLLKSSTASESDPIAKLLSSTALKTTQASEPIEVNASVGQTTSNPNH

PLPDTTSSVLQNLLDQTYPVV

**S7.C** BLP2 A-D sequence types

Sequence similarities among BLP2 encoded by ST1 AYE (A), ST419 AKAS(B),

ST23 BJAB0715 (C) and ST437 ATCC17978 (D) strains. Ig-fold motifs are in bold.

A MTRIIVASKEGLDVLQDGQLNKVVLNQPTIIQIGVSQKDIASMEKQGGSLVIHLKNGETIVLENFFNEATNTTEHSLVFPTEQGKFVEAQFDAQ

B MTRIIVASKEGLDVLQDGQLNKVVLNQPTIIQIGVSQKDIASMEKQGGSLVIHLKNGETIVLENFFNEATNTTEHSLVFPTEQGKFVEAQFDAQ

C MTRIIVASKEGLDVLQDGQLNKVVLNQPTIIQIGVSQKDIASMEKQGGSLVIHLKNGETIVLENFFNEATNTTEHSLVFPTEQGKFVEAQFDAQ

D ------------------------------------------MEKQGGSLVIHLKNGETIILENFFNEATNTTEHSLVFPTEQGKFVEAQFDAQ

******************:*********************************

A GKVIDYRGLNHVTDLAYTSTSPSAATMAVDNDPSFSMGNVLKAGLAVLAAEGLYLWAFDKDDKDDSPSTPDLIAPAAPTATLADDTV

B GKVIDYRGLNHVTDLAYTSTSPSTATMAVDNDPSFSMGNVLKAGLAVLAAEGLYLWAFDKDDKDDSPSTPDLIAPAAPTATLADDTV

C GKVIDYRGLNHVTDLAYTSTSPSTATMAVDNDPSFSMGNVLKAGLAVLAAEGLYLWAFDNDDKDDSPVN-VPTTAKPTEVKLADDAV

D GKVIDYRGLNHVTDLAYTSTSPSTATMAVDNDPSFSMGNVLKAGLAVLAAEGLYLWAFDNDDKDDSPVN-VPTTAKPTEVKLADDAV

***********************:***********************************:******* . :. .. ..****:*

A TVTGKTEANAKIYIKDAAGNTVASGVADASGNYTIKLDKPLVNGDKLNVIAQ **DAAGN** NSKVTVVTGTKDTIAPDVPQAQLSDDGS

B TVTGKSEANAKIYIRDLNGNTIASGVADASGNFTIKLDKPLTDGNKLKVVAE **DAAGN** ISKESEFIGKKDTIAPDAPQTQLSDDGS

C TVTGKAEANAKIYIKDLSGNVIASGNADASGNFTIKLDKPLTDGNKLNVSAQ NEGGK ESSAYSITGPKDTIAPDAPEAQLNEDGS

D TVTGKAEANAKIYIKDLSGNVIASGNADASGNFTIKLDKPLTDGNKLNVFAQ NGGGK ESIGAAITGTKDTIAPDAPQAQLSEDGS

*****:********:* **.:*** ******:********.:*:**:* *: **: .*:** * . * *******.*::**.:***

A LLTGKAEANAKITVYDATGKVLGTVFANKDGIYSLKLTPPLTSEAGGKVVAE **DAAGN** KSEEVKIIAGKDTIPPASPFVEVNKEGS

B LVTGKTEANVKITVYDATGKVLGTVFANKDGIFSLKLTPPLTSDAGGKVIAE **DVAGN** KSEPTKIIAGKDTFAPDVPLVEFNKEGT

C ILDGKTEPDAKVMIYDASGKFIDTVTASKEGKFSYKFTPPLT---GGKVVAI **DLAGN** ESKPTLIFAGKDTIPPKAPLLEVNKEGT

D ILDGKTEPDAMVFIYDASGKFIDTTKANSDGKFSYKFTPPLTSEAGGKVVAV **DKAGN** ESEPTKIIAGKDTFAPDIPLVEVNKEGT

:: **:*.:* : :***:**.:.*. *..:* :* *:***** ****** *** ***** :*: . *:*****:.* *::*.****:

A VIHGKTEANAKVQIKDADGKVIGSGTADAQGEFQITLSPALKEAQKGTVVVE **DAAGN** VSKPVEITPGFDSIAPDKPTVQINTDGT

B TVEGQTEPNAKVQIKDADGKVIGTGTANAQGEFQITLSSALKDSQKATIIVE **DAAGN** TSKPLEIKSGYDTLAPDKPTAQVNAEGT

C VIEGTAEANTKVYVKDADGNVIGTGTANAQGEFQITLSSALKDSQKATIIVE **DAAGN** TSKPLEIKSGYDTLAPDKPTAQINADGT

D MVEGKTEANAKVQIKDADGKVIGTGTANAEGEFQITLSSALKDSQKATIIVE **DAAGN** TSKPLEIKSGYDTIAPDKPTAQINADGT

:.* :*.*:** :*****:***:***:*:********.***::**.*::** ********* .***:**..*:*::******.*:*::**

A SVTGTAEANAKIEIKDTTGKVIGSGTADANGKFTISISPALTDNKHASVSAI **DNAGN** KSEVVDIVGTKDTTPPAKPILNSVDDDV

B SVTGTAEPNAKIEIKNSADKVIGTGTADANGKFTITISPALTDSSKGSVIAI **DSSGN** KSTALEIIGSDKDTIPPAKPSFSVYDDN

C TVTGTAEANAKIEIKNSAGKIIGTGTADANGKFTITISPALTDNKTADVIAI **DGAGN** SSGAFEVTGTKDTTPPDRPVFNKLENDN

D TVTGTAEANAKIQITIGT-KVIGTGTADANGKFTIAISPALTDNNKAKVTAI **DGAGN** SSVAFEVTGTKDTTPPDKPTFNSVYNDN

:******.****:*. : *:**:***********:*******.. ..* ** *.:** .* ..:: *:.. * * .: :*

A GAVKGAITAGSETDDARPKLTGSGEANATLTIYDNGVAIGVVTVTSG---RSWSFTFDKDLALGKHTITLTQTDAAGLTSEASSPFT

B GTSLKQLSANAETEDTTPKFTAKVESYATLTIYDNGVAISIIKIGDVGNSKNWSFTLDKELTFGKHTITLIQTDAAGLTSEVSSPFT

C GTTLKQILPNSETDDTTPKFTVKVESYATLTIYDNGVAISIIKIGDVGNSKNWSFTLDKELTFGKHTITLIQTDAAGLTSEVSSPFT

D GTTLKQIIPNSETDDTTPKFTVKVESHASLTIYDNGVAISMIEIGDLGKNTTWSFTLNKELSLGKHTITLIQKDAAGLPSEESASFT

*: : ..:**:*: **:* . *: *:**:*******.:: : . .****::*:*::******* *.*****..* *:.**

A FYVVAPKAASLSETSVDILSTEGPSLADSVGLHTLKVAQNTTTETNNPQ-KSVPLDDLLKSSTASESDPIAKLLSSTALKTTQASEPIEVNASVGQTTSNPNHPLPDTTSSVLQNLLDQTYPVV

B FYVVAPKAASLSETSVDTLSTEGPSLADSVGLHTLKVAQSTTTETNNPQ-KSVPLDDLLKSSTASESDPIAKLLSSTALKTTQASELIEVNASVGQTTSNPDHPLPDTTSSVLQNLLDQTYPVV

C FYVVAPKAASLSETSVDTLSTEGPSLADSVGLHTLKAAQSTSTETNNPQ-KSVPLDDLLKSSTASESDPIAKLLSSTALKTTQASEPIEVNASVGQTTSNADHPLPDTTSSVLQNLLDQTYPVV

D FYVVAPKVASTSEVVSSDVNEVVPSVTDSVGLNTLKLAQTTTNEMTSQQQKSVSLHDLLKSSSTDASDPIAKILSVTDSKPVVN-EQSEVNNIASDVTTNLEQLLPNTTSSPLQNILEQTYPVV

*******.** **. . :. **::*****:*** **.*:.* .. * ***.*.******::. ******:** * *.. * *** ..:.*:* :: **:**** ***:*:******
