# Supplementary material for: Biofilm-associated proteins: news from Acinetobacter
Source: BMC Genomics. 2015 Nov 14;16:933. doi: 10.1186/s12864-015-2136-6 (PMC4647330; doi:10.1186/s12864-015-2136-6)
Supplement: Additional file 8: — BLP1 empty chromosomal site A) DNA replacing BLP1 coding sequences in BLP1-negative strains. B) Alignment of BLP1 gene empty sites in A. baumannii and non-baumannii species. (DOCX 125 kb) [file 12864_2015_2136_MOESM8_ESM.docx]

**S8.A DNA replacing BLP1 coding sequences in BLP1-minus strains**

GAACAGTTGGCTGAATTCACTTCATATTGATTTGGTTTAGATGAGCTTAAATAATTGATTGTTTAAAAAAT

ATTCAAAAAAGAGAAAAATTTTTCTTAT

**S8.B Alignment of BLP1 gene empty sites in A. baumannii and non-Baumannii species**

Empty sites and flanking DNA are highlighted in grey and green respectively.

**Ba** CATGGGCATCTCCATAAAACAGCTATATATGATTTAACTCAGATTTATTCTTTAAAAATAGATCATCCTATTTTTGATATACATGCAGGTACAGCAACTTCTACTCGCTTATATCATCATAATCCAAATAGCTTTAATACAATTT

**No** CATGGTCATCTCCATAAAACGGCTATATATGATTTAACTCAAATTTACCATCTGGAAATAGATCATCCTATTTATGATATTCATGCGGGTACAGCAACTTCTACTCGTTTATATCATCATAATCCAAATAGCTTTAATACGGTTT

**Ha** CATGGGCATTTACATAAAGTTGCAGTGCATGATTTAAATCAAATTTTCGATTTAGGATTTGATCATCCGATTCTCGATATTCATGCAGGTACAGCGATATCCAATCGTGTACGTTTTGGATTGCCGAATAGTTTTAATGTGATTT

**Lw** CATGGGCATCTGCATCAACCGGCGATTTATGATTTGAATCAGATGTTCGGATTGGGGATGGATCATCCTGTATATGATGTACATGCAGGTACTTCTGCGTCAAATCGTCTACATAAAGATGAACCGAACAGCTTTAATCTGATTG

**Ra** CATGGCCATTTACATAAAACCGCGATCTATGATCTGACCCGACTGTATAATTTAAAAGTTGATCATCCTATTTATAATATTCATGCCGGTACTGCGACTTCCTGGCGCTTGCACAAGAACCTGCCGAACAGTTTTAACACTGTTT

**Ba** CAAATGAAGGAAAAATTCAGCATTATTGGTTTAAT [GAACAGTTGGCTGAATTCACTTCATATTGATTTGGTTTAGATGAGCTTAAATAATTGATTGTTTAAAAAATATTCAAAAAAGAGAAAAATTTTTCTTAT]

**No** CAAATACAGGAAAGATTCAGCATTATTGGTTTAAT [GAACAGCAGGCTGAATTCACTACAGATTGATTTGGTTTAGATGAGATTAAATATTTGATTGTTTAAAAAATATTCAAAAAACAGAAAAATTTTTCTTAT]

**Ha** TGAGCAATGGTGTGATTGAGCATTATTATTTTAAT [GAACAGTTTGAA-AAGTAGCGTAA-ATAGACTGAATTTA----AGATTCGTTTCGTGA-TGTTTGAAAAAAGAACAAAAAGTCGAAA--TTTTTTTTAT]

**Lw** ---ATGCAGGAAAAATTTCTCAATATTTATTCGAT [GAAG---TGGCACAAGCC--TTTATGTTGAA--GGCTGATCTGCGAATAAATAGACGAAAACCTATGATTCGTTAAAAAAAACCAATTTTTTTTCATTT]

**Ra** CAATATCTGGTGAAATTCTGCAATACTGGTTTAAT [GAAGAG--------AGTCAAT-CAT-TTGTTTTAGGACAGATGTGATTAAATAATTGATTGTTTAAAAAAAATACAAAAAT---ACAAATTTTTTTTAT

**Ba** TTCCCCTTGAAGCCATTTTTTTCATCCCCACAAAAGTGACATCTAAATATTTTGTTATTGCTCTGGATTAAAGCAATAACGATTAATCAAAAAGACTAAGTCTGATGGAGTTAATTATGAGCAACATTCGTCCATTACATGATCGCGT

**No** TTCCCCTTGAAGCCATTTTTTTCATCCCCACAAAAGTGACATCTAAATATTTTGTTATTGCTCTGGATTAAAGCAATAACGATTAATCAAAAAGACTAAGTCTGATGGAGTTAATTATGAGCAACATTCGTCCATTACATGATCGCGT

**Ha** TTCCCCTTGAAGCCTTTTTTTTTAACCCCACAAAAGTGGCATATCAAAATTTTGTTATTGCTCTGGAGTA--GCAATGCAAATTAATCAAAAAGACTAAGTCTGATGGAGTTAAATATGAGCAACATTCGTCCATTACATGATCGCGT

**Lw** TTGCCCTTGAAGCGTTTTATTCCATCTCCATAAAAGTGACATCAAAAATTTTTGATGATGCCATGGAATAAAGTCGTCATCAGTGATAAAAAAGACTTAGTCTGATGGAGTTATTTATGAGCAACATTCGTCCATTACATGACCGCGT

**Ra** TTCCCCTTGAAGCGTTTTTTTCTATCCCCACAAAAGTGTCATCAAAACACTTTGTTATTACTTAGACTTAAAGTAATAACGATTACTTAAAAAGACTTAGTCTGATGGAGTTAATTATGAGCAATATTCGTCCATTACATGACCGTGT

**Ba,** *A. baumannii***: No**, *A. nosocomialis* (AMJH strain); **Ha**, A. *haemolyticus* (APQR strain); **Lw**, *A. lwoffii* (AYHO strain);

**Ra**, *A. radioresistens* (APQE strain)
