# Supplementary material for: Biofilm-associated proteins: news from Acinetobacter
Source: BMC Genomics. 2015 Nov 14;16:933. doi: 10.1186/s12864-015-2136-6 (PMC4647330; doi:10.1186/s12864-015-2136-6)
Supplement: Additional file 10: — OmpA sequence variants. Organization of SURP-1. (DOCX 117 kb) [file 12864_2015_2136_MOESM10_ESM.docx]

S9.Changes in the aa composition of the interval 35-183 of ompA (Accession number AY485227).

ompA FQDSQHNNGGKDGNLTNGPELQDDLFVGAALGIELTPWLGFEAEYNQVKGDVDGASA-GAEYKQKQINGNFYVTSDLITKNYDSKIKPYVLLGAGHYKYDFDGVNRGTRGTSEEGTLGNAGVGAFWRLNDALSLRTEARATYNADEEFWNY

ST25 AEPA WQDSEHNNN----KLTDHAELQDDLFVGAGLGVELTPWLGFEAEYNQVKGDLDGTGVQGAEYKQKTIAGNFYATSDLITKNYDSKFKPYVLLGAGQTKTEFDGIYE-----DKKDTIGNAGVGAFYRLNDALSLRTEARGTYDFDEKYWRY

ST25 JEVX WQDSEHNNN----KLTDHAELQDDLFVGAGLGVELTPWLGFEAEYNQVKGDLDGTGVQGAEYKQKTIAGNFYATSDLITKNYDSKFKPYVLLGAGQTKTEFDGIYE-----DKKDTIGNAGVGAFYRLNDALSLRTEARGTYDFDEKYWRY

ST25 AEPM WQDSEHNNN----KLTDHAELQDDLFVGAGLGVELTPWLGFEAEYNQVKGDLDGTGVQGAEYKQKTIAGNFYATSDLITKNYDSKFKPYVLLGAGQTKTEFDGIYE-----DKKDTIGNAGVGAFYRLNDALSLRTEARGTYDFDEKYWRY

ST25 AMHN WQDSEHNNN----KLTDHAELQDDLFVGAGLGVELTPWLGFEAEYNQVKGDLDGTGVQGAEYKQKTIAGNFYATSDLITKNYDSKFKPYVLLGAGQTKTEFDGIYE-----DKKDTIGNAGVGAFYRLNDALSLRTEARGTYDFDEKYWRY

ST25 APOU WQDSEHNNN----KLTDHAELQDDLFVGAGLGVELTPWLGFEAEYNQVKGDLDGTGVQGAEYKQKTIAGNFYATSDLITKNYDSKFKPYVLLGAGQTKTEFDGIYE-----DKKDTIGNAGVGAFYRLNDALSLRTEARGTYDFDEKYWRY

ST25 CBSG WQDSEHNNN----KLTDHAELQDDLFVGAGLGVELTPWLGFEAEYNQVKGDLDGTGVQGAEYKQKTIAGNFYATSDLITKNYDSKFKPYVLLGAGQTKTEFDGIYE-----DKKDTIGNAGVGAFYRLNDALSLRTEARGTYDFDEKYWRY

ST25 AHAI WQDSEHNNN----KLTDHAELQDDLFVGAGLGVELTPWLGFEAEYNQVKGDLDGTGVQGAEYKQKTIAGNFYATSDLITKNYDSKFKPYVLLGAGQTKTEFDGIYE-----DKKDTIGNAGVGAFYRLNDALSLRTEARGTYDFDEKYWRY

ST25 JEWM WQDSEHNNN----KLTDHAELQDDLFVGAGLGVELTPWLGFEAEYNQVKGDLDGTGVQGAEYKQKTIAGNFYATSDLITKNYDSKFKPYVLLGAGQTKTEFDGIYE-----DKKDTIGNAGVGAFYRLNDALSLRTEARGTYDFDEKYWRY

ST25 AFDL WQDSEHNNN----KLTDHAELQDDLFVGAGLGVELTPWLGFEAEYNQVKGDLDGTGVQGAEYKQKTIAGNFYATSDLITKNYDSKFKPYVLLGAGQTKTEFDGIYE-----DKKDTIGNAGVGAFYRLNDALSLRTEARGTYDFDEKYWRY

ST113 AMHF WQDSEHNNN----KLTDHAELQDDLFVGAGLGVELTPWLGFEAEYNQVKGDLDGTGVQGAEYKQKTIAGNFYATSDLITKNYDSKFKPYVLLGAGQTKTEFDGIYE-----DKKDTIGNAGVGAFYRLNDALSLRTEARGTYDFDEKYWRY

ST113 AMHG WQDSEHNNN----KLTDHAELQDDLFVGAGLGVELTPWLGFEAEYNQVKGDLDGTGVQGAEYKQKTIAGNFYATSDLITKNYDSKFKPYVLLGAGQTKTEFDGIYE-----DKKDTIGNAGVGAFYRLNDALSLRTEARGTYDFDEKYWRY

ST133 AVST WQDSEHNNN----KLTDHAELQDDLFVGAGLGVELTPWLGFEAEYNQVKGDLDGTGVQGAEYKQKTIAGNFYATSDLITKNYDSKFKPYVLLGAGQTKTEFDGIYE-----DKKDTIGNAGVGAFYRLNDALSLRTEARGTYDFDEKYWRY

ST138 JFDB WQDSEHNNN----KLTDHAELQDDLFVGAGLGVELTPWLGFEAEYNQVKGDLDGTGVQGAEYKQKTIAGNFYATSDLITKNYDSKFKPYVLLGAGQTKTEFDGIYE-----DKKDTIGNAGVGAFYRLNDALSLRTEARGTYDFDEKYWRY

ST412 AMZT WQDSEHNNN----KLTDHAELQDDLFVGAGLGVELTPWLGFEAEYNQVKGDLDGTGVQGAEYKQKTIAGNFYATSDLITKNYDSKFKPYVLLGAGQTKTEFDGIYE-----DKKDTIGNAGVGAFYRLNDALSLRTEARGTYDFDEKYWRY

ST519 JEYH WQDSEHNNN----KLTDHAELQDDLFVGAGLGVELTPWLGFEAEYNQVKGDLDGTGVQGAEYKQKTIAGNFYATSDLITKNYDSKFKPYVLLGAGQTKTEFDGIYE-----DKKDTIGNAGVGAFYRLNDALSLRTEARGTYDFDEKYWRY

ST529 JEWL WQDSEHNNN----KLTDHAELQDDLFVGAGLGVELTPWLGFEAEYNQVKGDLDGTGVQGAEYKQKTIAGNFYATSDLITKNYDSKFKPYVLLGAGQTKTEFDGIYE-----DKKDTIGNAGVGAFYRLNDALSLRTEARGTYDFDEKYWRY

Organization of SURP-1. The sequence of the AYE ORF 3068 is shown

**NH2 region**

MFIKSILSSITSIIPLPENSNTSSNL

**Repetitive region**

GNGSGDGLLNGISSGNGEHNYGIGNGIADDASITAPITIPLNLSGNSITLIGNSSSSSVNSSPTTTSNNVNDNDVTNNGNGSTIGSGT

GNGSGDGLLNGAASGNGEHNYGIGNGIADDASITAPLSIPINLAGNSITLIGDSSSSSVNNSATNTSNTVNDNDTTYNGNGSGG---

GNGSGDGLLNGIGSGNG--NYGIGNGIADDASITAPITLPINLSGNSITLIGNSSASSVNSSPTTTSNTVNDNDTTYNGNGTGDSGVSALGGS

GNGSGDGAGNGIASGNGEHNYGIGNGNGDDVDITAPITGVLNISGNSFTLIGNSSSSSVNTAPTTTSNTVNDNDTIDNGNSGGGSGS

GNGSGDGLLNGAASGNGEHNYGIGNGNGDDVDITAPITGVFNFSGNSFSIIGNSSSSSINTAPTTTTNTVNDNDVTDNGNDGGGLVGGSS

GNGSGDGLLNGAASGNGEHNYGIGNGNGDDADFTFPLTGVLNFSGNSLSGFGSSSSDSVNVAPTTATNTVNDNDTIDNANTGGLGDGS

GNGSGDGLLNGAASGNGEHNYGIGNGNGDDADFTLPFTGGLNILGNALSGIGGSSTDSINISPTTTSNTVNDNDTTNNGNTSGGVIGSGDS

GNGSGDGLLNGISSGNGEHNYGIGNGNGDDVDVVAPITTPLNVLGNSFSFIG

**COOH region**

GEGTGDILGPITGIIGGIGGDGDILSPITGIIGGIGGDGDILSPITGIIGSIGGIGGDLGDNPLTGIIQSGIDVLQNLES

LKTGLINTGIDTIAGTIIGVFPDAEHPVGDFADLGKLLFETSRDSVNGTLEAISDLAGADLEGASGSITGVIDTLITNGS

TASTIIQHIVGDDLVTENGGLLGSITTIIGGVDSGDGGLLGGLDGLISINYGDSDNSNSIDVEDILGNILGSVGSNQGIA

VGEPDPTGGSLIHTISLNTVNQLTDQLLHALPTV
